# Effects of converting cropland to grassland on greenhouse gas emissions from peat and organic-rich soils in temperate and boreal climates: a systematic review

**DOI:** 10.1186/s13750-024-00354-1

**Published:** 2025-01-19

**Authors:** Alena Holzknecht, Magnus Land, Jacynthe Dessureault-Rompré, Lars Elsgaard, Kristiina Lång, Örjan Berglund

**Affiliations:** 1 Department of Soil and Environment, Swedish University of Agricultural Sciences, Box 7014, 750 07 Uppsala, Sweden; 2 Formas, Box 1206, 111 82 Stockholm, Sweden; 3 Département des sols et de Génie Agroalimentaire, Faculté des sciences de l’Agriculture et de l’alimentation, Université Laval, 2325 Rue de l’Université, Québec, QC G1V 0A6 Canada; 4 Department of Agroecology, Aarhus University, Blichers Allé 20, 8830 Tjele, Denmark; 5 Natural Resources Institute Finland (Luke), Latokartanonkaari 9, 00790 Helsinki, Finland

**Keywords:** Gas fluxes, Climate change, Mitigation, Land-use, Peat soils, Policy

## Abstract

**Background:**

To align with climate goals, greenhouse gas (GHG) emissions from agriculture must be reduced significantly. Cultivated peatlands are an important source of such emissions. One proposed measure is to convert arable fields on peatlands to grassland, as the Intergovernmental Panel on Climate Change (IPCC) default emission factors (EF) for organic soils are lower from grasslands. Yet, these EFs are based on limited data with high variability and comparisons are difficult due to differences in climate, soil properties, and crop management. This systematic review synthesizes available evidence on the effects of converting cropland to grassland on GHG emissions from peat and organic-rich soils in temperate and boreal climates using data from comparable fields.

**Methods:**

Literature was searched using five bibliographic databases, four archives or search engines for grey literature, and Google Scholar. Eligibility screening was performed in two steps on (1) title/abstract, with consistency among reviewers assessed by double-screening 896 articles, and (2) full text screened by two reviewers. Eligible articles were critically appraised independently by at least two reviewers. Disagreements were reconciled through discussions. Data and key metadata are presented in narrative synthesis tables, including risk of bias assessments. Meta-analyses comparing grasslands with croplands were performed using raw mean difference as the effect size.

**Review findings:**

A total of 10,352 unique articles were retrieved through the literature searches, and 18 articles including 29 studies were considered relevant to answer the review question. After critical appraisal, it was concluded that two articles reported the same data, so a total of 28 studies, comprising 34 comparisons were included in the systematic review. Most of the included studies were conducted in the Nordic countries and Germany, one in Belarus and one in Canada. A meta-analysis was conducted on 24 studies pairing cropland and grassland sites. No significant differences in carbon dioxide (CO_2_) or methane (CH_4_) emissions were found. Emissions of nitrous oxide (N_2_O) from grasslands were found to be 7.55 kg ha^−1^ y^−1^ lower than from cropland, however the sensitivity analysis showed that the difference was not robust, making it uncertain whether conversion from cropland to grassland has a significant effect on N_2_O emissions from organic soils. The difference was also smaller when root crops were excluded from the comparator group. Further, net ecosystem exchange (NEE) of CO_2_ and net ecosystem carbon balance (NECB) were higher in grasslands compared to croplands in cases where the grasslands were fertilized.

**Conclusions:**

This systematic review underlines the ambiguity of GHG emissions from peatlands and their relationship to land use. Our understanding of the factors influencing emissions from these soils remains incomplete, and the specific impact of land use on emissions is still unclear. CO_2_ emissions represent a major part of the climate impact of cultivated peat soils, so the data analyzed allow to draw the conclusion that a conversion from arable to grassland would not lead to large benefits in terms of GHG emissions, especially if root crops are not part of the arable crop rotation, or the grassland is fertilized.

**Graphical abstract:**

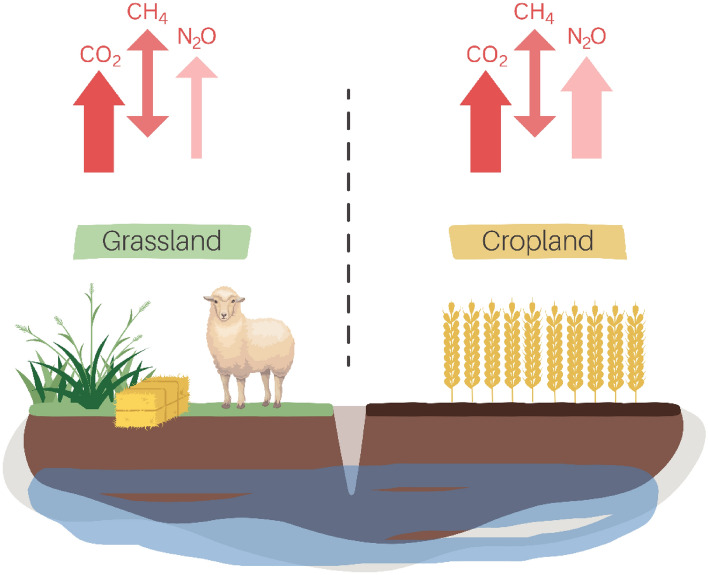

**Supplementary Information:**

The online version contains supplementary material available at 10.1186/s13750-024-00354-1.

## Background

The agricultural sector plays a significant role in global greenhouse gas (GHG) emissions, accounting for approximately 12% of total emissions, or 23% if also emissions reported under the category “Land Use, Land Use Change and Forestry” (LULUCF) are included [1]. Peatlands, of which about 80% are located in boreal and temperate regions, are of particular interest due to their significant contribution to GHG emissions [2]. In the northern European countries, a considerable proportion of agricultural land consists of drained peat soils, making them significant sources of carbon dioxide (CO_2_) and nitrous oxide (N_2_O) emissions [3]. Given the international ambition to reduce global GHG emissions under the Paris Agreement and the need to strengthen the carbon sink of the land use sector for carbon neutrality [4], several countries have already set national targets for agricultural emissions [5]. It has been recognized that organic soils can both contribute to emission reductions and act as a carbon sink. Thus, several proposed legal acts aim to support measures to reduce their GHG emissions also outside of the agricultural subsidy schemes. For example, the EU has addressed drained peat soils in the suggested certification framework for carbon removals [6] and in the taxonomy for sustainable funding [7].

Microbial processes produce and consume the greenhouse gases CO_2_, methane (CH_4_), and N_2_O in soil environments but these processes are strongly regulated by environmental conditions [8]. While our understanding of the basic mechanisms behind GHG production and turnover in soils is relatively robust, the specifics of how different agricultural practices influence these processes in peat soils remain unclear. The estimation of the quantity and direction of fluxes of these gases is further complicated by soil properties like organic matter content and quality, nutrient availability, soil water content and peat degradation status [9–13].

There is a need to strengthen the scientific evidence base for mitigation measures to facilitate cost-effective policies. Rewetting drained organic soils is a well-recognized GHG mitigation strategy [14]. However, it is costly and renders arable organic soils unsuitable for food production, potentially leading to GHG leakage by shifting cultivation elsewhere. Suggested measures that allow peat soils to be cultivated while potentially reducing GHG emissions include growing different grasses [15], adding sand [16] or lime [17] to the soil, different cultivation systems or intensities [17, 18], water management [19], abandonment [20] and adding polyphenols [21]. The Intergovernmental Panel on Climate Change (IPCC) emission factors for temperate and boreal regions suggest that the CO_2_ emissions are 20–30% lower in grasslands compared to croplands [22], which makes leys an attractive GHG mitigation solution for policy makers. For farmers, changing the crop type from annual to perennial, however, may have practical constraints and economic implications.

The default IPCC emission factors available for reporting emissions from organic soils are based on compilations of scientific publications [22]. As the data sets are limited, the comparisons of cultivation practices are complicated by high variability in climate and weather conditions, monitoring time and crop management. In these research compilations, sites with grass were not always permanent grasslands and the treatments were compared without taking into account that the landowner may have selected certain types of fields for annual and perennial management based on the site properties. It is therefore possible that different peat quality or poorer drainage on the average may partly explain the lower emissions from grasslands and that a change from arable cultivation to grassland on a fertile and well-drained site may not result in the predicted emission reduction.

In this article, we compare GHG emissions from organic soils in annual and perennial cropping systems using a carefully selected dataset compiled from studies with an experimental design where both management options were present under comparable conditions and at the same time. We outline the current understanding of GHG emissions from cultivated peat soils, highlight key challenges, and discuss the role of stakeholders in shaping the research agenda. Finally, we highlight implications for research and policy that aim to contribute to a better understanding of mitigation strategies to reduce GHG emissions from peat soils in agriculture.

The systematic review was proposed by the Swedish Board of Agriculture. Although they were involved in framing the review question and were informed about preliminary results during the conduct of the review, they were not directly involved in the work. All decisions, analyses, and conclusions were made independently by the review team.

## Objective of the review

The main objective of this review is to determine whether changing land use on peat soils from arable production to grassland reduces GHG emissions from the soil. This study specifically aimed at finding studies where cropland and grassland sites were close to each other and had comparable characteristics, as lack of comparability was the main constraint found in most studies on this topic.

While the research question originates from a Nordic perspective, data from various regions worldwide were considered if they met our eligibility criteria. We expect the findings from this review to be relevant and applicable to all regions with comparable agricultural practices within the boreo-temperate climate zones.

The review question we aim to answer with this systematic review is:”What is the effect of permanent grasslands on the flux of greenhouse gases from agricultural organic soils?”. The PICO (Population, Intervention, Comparator and Outcome) elements of the question are:

- *Population*: Organic soils on agricultural land in temperate and boreal climate zones. Such organic soils are often drained peatlands, but other origins, such as lake beds, may occur.
- *Intervention*: Using land for grazed or ungrazed, permanent or cultivated grassland (ley) or setting land aside from agricultural production (perennial green fallow) without any attempt to raise the groundwater level. Rewetted grasslands are thus not included. Growing woody energy crops is not an eligible intervention or comparator. Growing grass-like energy crops is an eligible intervention.
- *Comparator*: Using land for various crop rotations involving annual crops. Land uses may be categorised regarding tillage, fertilisation, and other management practices.
- *Outcome*: Flux of CO_2_, N_2_O, or CH_4_.

## Methods

This systematic review followed the Guidelines and Standards for Systematic Reviews of the Collaboration for Environmental Evidence [23]. The methods of this review adhered to a published systematic review protocol [24] with a few minor deviations reported below.

### Deviations from the protocol

We refined the description of eligible CO_2_ response variables to ensure greater accuracy and relevance to the review objectives. We have now included net ecosystem exchange (NEE), net ecosystem carbon balance (NECB), ecosystem respiration (R_eco_) and soil respiration (R_soil_) to describe CO_2_ fluxes. This adjustment was necessary to capture a more precise range of data pertinent to GHG emissions from agricultural organic soils. Secondly, we opted not to use log response ratios as effect size in our analysis, as described in the protocol, because the presence of negative values in the data set made this approach impossible. Thirdly, we decided not to use Research Rabbit to find further sources, as after some initial trials it did not yield any additional results on top of snowballing through references in review articles.

### Search for articles

#### Search sources

As mentioned in the review protocol, we searched the bibliographic databases Scopus, Web of Science Core Collection, CAB Abstracts, ProQuest Natural Science Collection, and Directory of Open Access Journals (DOAJ). Additionally, we searched for grey literature using BASE (Bielefeld Academic Search Engine), SwePub, Finna, ProQuest Theses and Dissertations, and Google Scholar. Search dates and other details can be found in Additional file 1. We also performed a snowballing process, i.e., we looked for additional relevant articles in the reference list of all included papers and in those that were marked as “relevant review” in the title and abstract screening. Here, no additional relevant articles were found.

#### Search terms and strings

The search strings used for bibliographic databases consist of three substrings relating to peat soil (population), grassland (intervention), and GHG emission (outcome), respectively. While the words within each string are combined with an OR operator, the strings are combined with an AND operator (see Table 1). Adaptations of the search string for each database and search engine, and details on searched fields are shown in Additional file 1.

**Table 1:** Search terms used for searches in bibliographic databases. The asterisk is a wildcard representing any number of characters

No	Substring
1	“organic soil" OR "organic soils" OR peat* OR histosol* OR "muck sediment" OR "muck sediments" OR "muck soil" OR "muck soils" OR gyttja OR moorsh* OR wetland* OR turf* OR coprogenous OR muskeg OR suo OR mud OR muds OR swamp OR swamps OR lowland* OR fen OR fens OR mire OR mires OR marsh* OR morass OR quag* OR gley* OR "carbon rich" OR "black soil" OR "black soils" OR bog* OR "high organic carbon" OR hydromorphic
2	grass OR grassland* OR ley* OR fallow OR pasture OR forage OR perennial* OR mesocosm* OR lysimeter* OR semifield* OR legume* OR pulse* OR alfalfa* OR lupin* OR bean* OR lentil* OR clover* OR meadow* OR timothy OR set-aside OR setaside OR pea OR peas OR crop* OR graz*
3	"greenhouse gas" OR "greenhouse gases" OR "carbon dioxide" OR CO2* OR "carbon emission" OR "carbon emissions" OR "nitrous oxide" OR "nitrous oxides" OR N2O OR "laughing gas" OR methane OR CH4 OR "global warming potential" OR GHG* OR "net ecosystem exchange" OR "net ecosystem production" OR respiration OR "carbon balance" OR "trace gas" OR "trace gases" OR NEE OR NEP OR "carbon turnover" OR "eddy covariance" OR "dinitrogen oxide" OR "dinitrogen monoxide" OR "marsh gas"

#### Search limitations

Publications in languages other than English are relevant, as many countries fall within the geographical scope of this review. We conducted additional searches for grey literature in the languages French, Finnish, Swedish, Danish and German, as these languages are spoken by the authors of this publication and represent some major areas where we expected to find studies. The foreign language search strings used can be found in Additional file 1. However, other languages that were not searched may have yielded relevant results that are not included in this review. No restrictions were applied to the time period or document type of the articles.

#### Estimating the comprehensiveness of the search

A list of benchmark articles, including articles relevant to the review question, was compiled by the review team and used to test the comprehensiveness of the selected search strings [24]. All but one of the articles indexed in at least one of the searched bibliographic databases were retrieved by our search strings. The missing article [25], in Danish, has only a brief English abstract and did not fully meet our outcome inclusion criteria, so no adjustments were made. Google Scholar searches in English retrieved all of the benchmark grey literature except one German thesis [26]. Although the thesis appears in search results when using its title, it was not ranked among the top 300 results and we judged it unfeasible to adjust the search strategy any further.

#### Search results

All records including their bibliographic information were exported to EndNote 20 where deduplication was performed. The unique records were then exported to EPPI reviewer [27] where some additional duplicates were identified and removed. EPPI reviewer was also used to conduct the article screening process.

### Article screening and study eligibility criteria

#### Screening process

First, the repeatability of the screening process was tested by screening 600 articles on title and abstract level in two groups of three reviewers, evaluating the eligibility criteria and resolving disagreements. A further subset of 300 articles was double screened at the title and abstract level by two reviewers (AH and ML) reassessing their consistency and finally the remaining articles were screened in single mode. It was decided at this stage to exclude articles that did not mention any GHG fluxes in the title or abstract. Included articles were further double screened at full text level, with reasons for exclusion coded at both levels as “population”, “intervention/exposure”, “comparator”, “outcome”, “study design”, or “review article”. In most cases several reasons applied, but only the first apparent reason was marked. The inclusion of the final articles was discussed in several feedback rounds with the entire review team. To avoid potential conflicts of interest, members of the review team did not participate in decisions regarding the inclusion or validity assessment of studies in which they were authors. After each round of double screening, Cohen’s kappa values were calculated to check the consistency between the reviewers. However, no threshold level was defined a priori to decide whether the consistency was acceptable.

#### Eligibility criteria

The studies were screened based on the following criteria: population, intervention, comparator, outcome, and study design.

- Eligible population(s) or subject(s)

To qualify for this review, articles had to include organic soils on agricultural land in temperate and boreal climate zones. In the review protocol it was planned to differentiate between “true” peat soils and organic soils with lower soil organic carbon (SOC) concentration based on varying definitions of organic soils. “True” peat soils were defined as Histosols [28] or soils with a SOC concentration greater than 12% and a peat depth greater than 30 cm. Shallow and/or low SOC peat soils were defined as having 6–12% SOC and a depth greater than 10 cm. However, due to the limited number of relevant articles identified, it was not feasible to further subdivide them into the proposed categories. The limiting factor was the lack of data for 6–12% SOC soils in the eligible articles. Instead, we performed a meta-regression with SOC concentration as the independent variable.

The climate zones considered in this study are Cfb (warm temperate, fully humid, warm summers) and Dfa, Dfb, and Dfc (snow climate, fully humid) according to the Köppen climate classification [29]. Since the climate zone is not always reported in studies and classifications may change over time, the eligibility of all studies was based on the current classification according to the World Map of the Köppen-Geiger Climate Classification published at https://koeppen-geiger.vu-wien.ac.at/.

- Eligible intervention(s) or exposure(s)

To be included, articles must include grazed or ungrazed, permanent or cultivated grassland or land set aside from agricultural production. The grassland must have been without tillage for at least three years in accordance with the regulations of the Swedish Board of Agriculture that require a minimum of three continuous years of ley to receive environmental payments [30]. Rewetting of peatland or cultivation of woody energy crops are not eligible interventions. Growing grass-like energy crops is an eligible intervention, as these may have similar characteristics to other grassland types.

- Eligible comparator(s)

Included studies must use the land for annual cropping. The specific crops or crop rotations are documented as potential effect modifiers. Each study must include a cropland comparison where outcomes (i.e., GHG fluxes) are measured using the same method at the same time under comparable peat soil conditions, climate, location, and other relevant factors, to ensure consistency and comparability of results.

- Eligible outcomes

Included studies must report the flux of CO_2_, N_2_O, or CH_4_, or a combination of these, and fluxes must be measured directly using methods such as dark or transparent chambers, eddy covariance measurements, or concentration gradient techniques. Studies estimating gas fluxes based on indirect measures, such as soil subsidence or changes in soil organic carbon stocks, were not eligible. The flux of CO_2_ may be reported as NEE, NECB, R_eco_, or R_soil_. As these response variables have different meanings we have treated them separately in the data synthesis section.

- Eligible types of study design

No initial limitations were imposed on study characteristics like study duration, number of replicates, or sampling frequency, in anticipation of a limited number of eligible studies. Rather than specifying numerical restrictions, we ensured that each article clearly described a system capable of addressing the review question. Eligibility and data quality were rigorously assessed during the study validity evaluation. Mesocosm studies were deemed eligible, provided that the mesocosms were sufficiently large (greater than approximately 0.5 m^2^) and contained soil that was sufficiently undisturbed to mimic a full-scale grassland. Modelled data were not included, though studies were tracked back to check for the input (model validation) data.

### Study validity assessment

The critical appraisal process was conducted to ensure the rigor and reliability of the included studies. We used the *Collaboration for Environmental Evidence Critical Appraisal Tool Version 0.3 (Prototype)* [31] and adjusted it to the needs of this study. Our adjusted template can be found in the review protocol [24].

For the critical appraisal itself, the subject experts of the review team (ÖB, JDR, LE, KL) initially were calibrated to standardize the evaluation criteria and ensure consistency across the assessments. This calibration included training sessions and discussions to agree on the definitions and thresholds for study validity and relevance. The studies were then subjected to pair-wise comparisons by the experts. Each study was independently evaluated by at least two experts to minimize bias and increase the reliability of the assessment. Discrepancies between evaluations were discussed and resolved by consensus. The outcomes of the critical appraisal process were systematically recorded in the adjusted version of the critical appraisal tool. For the meta-analysis we also conducted a sensitivity analysis with and without the articles that had an unclear risk of bias.

### Data coding and extraction strategy

In this systematic review we distinguish between articles, studies, and comparisons. Each published article may contain more than one study, where a study is defined as an investigation at a specific location and time, using a specific gas flux measurement technique. Each study, in turn, may encompass several comparisons, where a comparison is a contrast between a specific grassland and a specific cropland with a specific crop rotation. A study may thus provide data for several comparisons, and a comparison may provide data for several outcome measures or response variables.

Study metadata were recorded in a pre-designed Excel datasheet (Additional file 2). Extracted metadata include bibliographic details, study area details (e.g., coordinates, climate zone), grassland and cropland details (e.g., SOC concentration, total soil nitrogen (N), fertilization, pH, bulk density, drainage depth, groundwater level, crop rotation), sampling details (e.g., start and end of sampling period, sampling frequency, gas sampling technique, chamber size and position), and outcome details (e.g., measured gases and response variable for CO_2_ flux).

Outcome data were recorded in a separate Excel file for each article and for all reported time points (Additional file 2). All data were then combined in a single Excel data sheet and converted to the same units. In cases where outcome data were reported in graphical figures, we used WebPlotDigitizer [32] to extract the data.

### Potential effect modifiers/reasons for heterogeneity

In the review protocol, we identified several potential effect modifiers and reasons for heterogeneity. These were agreed upon in consultation with the protocol development team and included soil parameters such as SOC concentration, moisture, pH, bulk density, degree of decomposition or peat depth, drainage or groundwater table depth, time since drainage, time since conversion to annual cropland and ley/perennial fallow, tillage practices, applied fertilizers and crop residues, and measurement methods. These modifiers were chosen based on their theoretical impact on the outcomes and their relevance to the external validity of our results.

### Data synthesis and presentation

All studies included in the review are included in narrative synthesis tables (see review findings), including a metadata table and a data table. The metadata table provides bibliographic information, information on study characteristics, risk of bias assessments, external validity assessments, and whether study results were used in meta-analysis. If study results were not used in the meta-analysis, a reason is given. The data table shows data (results) extracted from the included articles, together with calculated effect sizes and key metadata.

Quantitative synthesis through meta-analysis was performed when at least five studies reporting the same response variable were available. The raw mean difference (D) is used as effect size, expressed in Mg ha^−1^ y^−1^ for CO_2_ response variables (NEE, NECB, R_eco_) and kg ha^−1^ y^−1^ for CH_4_ and N_2_O flux. No meta-analysis was performed for R_soil_ as less than five comparisons were reported for that response variable. For studies with independent treatment groups the raw mean difference was calculated as

1 $$D={\overline{X} }_{I}-{\overline{X} }_{C}$$

where $\overline{X }$ is mean and *I* and *C* denote intervention group and control group, respectively. In line with most included studies in this systematic review, we define positive fluxes as fluxes from the soil to the atmosphere. Thus, negative values of D imply that the GHG flux to the atmosphere is lower from permanent grasslands than from croplands. The within-study variance estimates (${V}_{D}$) were calculated as:

2 $${V}_{D}=\frac{{n}_{I}+{n}_{C}}{{n}_{I}{n}_{C}}{S}_{pooled}^{2}$$

where *n* is number of replicates and *S* is standard deviation. Equation 2 assumes that the two population standard deviations are similar, and can be pooled as:

3 $${S}_{pooled}^{2}=\sqrt{\frac{\left({n}_{I}-1\right){S}_{I}^{2}+\left({n}_{C}-1\right){S}_{C}^{2}}{{n}_{I}+{n}_{C}-2}}$$

Some studies investigated multiple control treatments (crop rotations) and one shared intervention treatment (permanent grassland). Comparisons between the shared intervention and the different control treatments are not independent. To account for the non-independence of sampling variances, we constructed a variance covariance matrix [33] where the group weights were assumed to be identical across treatments (group sample sizes are equal), which implies a correlation of rho = 0.5 [34]. Moreover, effect sizes from studies conducted at the same site (and soil) may not be independent even if there is a separate intervention group for each control group, and to account for non-independence among such effect sizes we used a multilevel random-effects model nested around study location. In cases where studies reported results from multiple timepoints (years) we used only data from the last timepoint. The flux of CO_2_ has been reported in many forms, but unless stated otherwise we have used the results for NEE, NECB, and R_eco_ in separate analyses. In each meta-analysis, we have thus used only one observation per comparison and one response variable per gas flux (outcome).

Meta-analysis was carried out in R (version 4.2.0) using the metafor package [34]. The model was fitted via restricted maximum likelihood (REML) estimation of variance components. We used the *I*^*2*^-statistic [35] to estimate the proportion of the observed variance that reflects heterogeneity (i.e., differences between studies not explained by sampling variance). However, as we used a multi-level model, we used the method suggested by Nakagawa et al. [33] to decompose *I*^*2*^_*total*_ into variance due to differences between study locations (*I*^*2*^_*loc*_) and variance due to differences within study locations (*I*^*2*^_*eff*_). To assess the risk of publication bias, funnel plots and Egger’s regression tests were employed. Essential parts of the R code are shown in Additional file 3.

## Review findings

### Review descriptive statistics

**Fig. 1:**
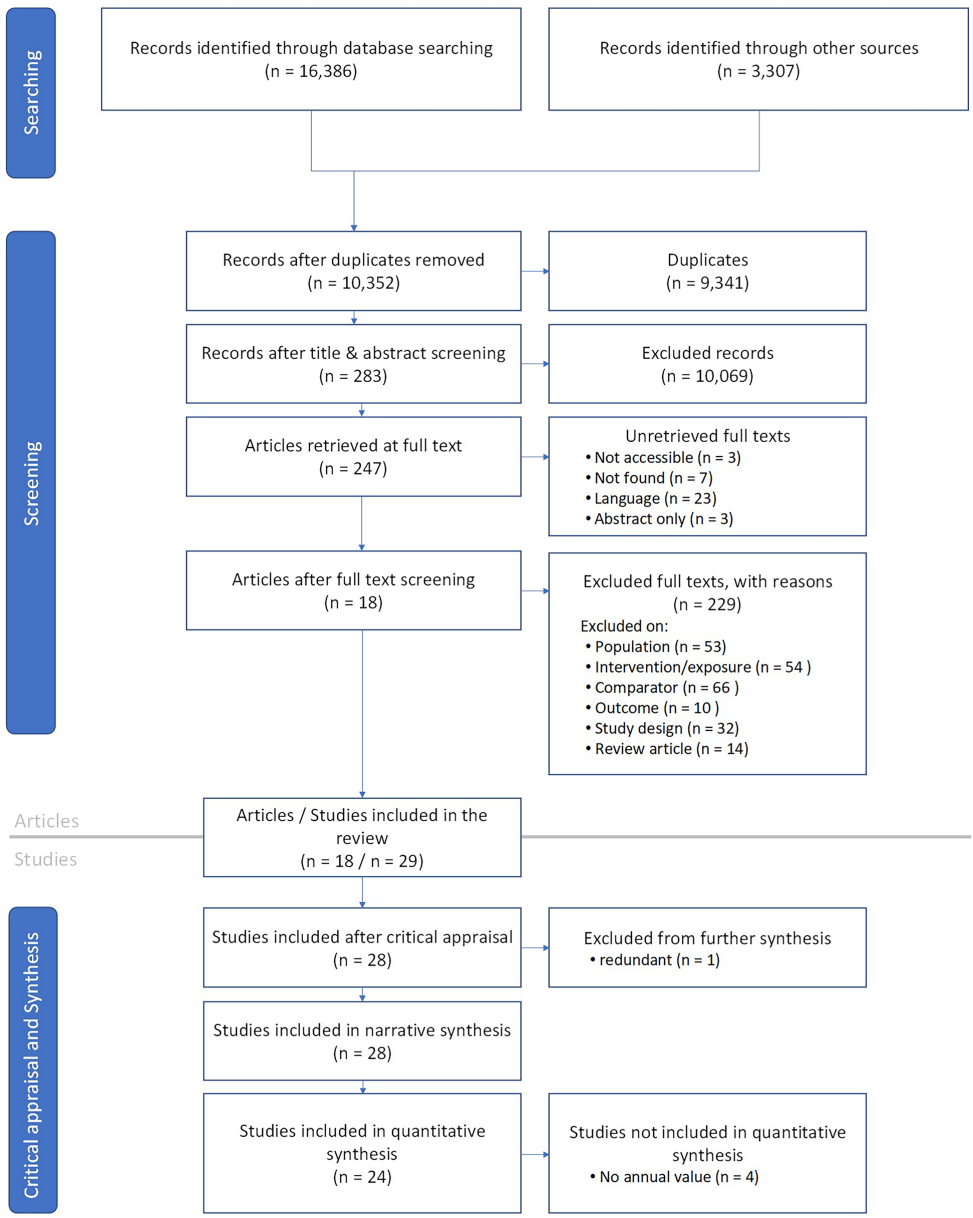
Literature screening results shown in a ROSES flow diagram (https://www.roses-reporting.com/)

The literature searches in bibliographic databases resulted in 16,386 records while the searches in additional sources resulted in 3,307 records (see Additional file 1). After deduplication, 10,352 unique records remained (Fig. 1). After screening titles and abstracts, 283 records were retained of which 247 could be retrieved in full text. Screening of those resulted in 18 relevant articles. During critical appraisal it was concluded that one of those articles [36] reported the same data as another article [37]. The included records reported data for 28 studies, with a total of 34 comparisons between permanent grassland and crop rotation. A list of unretrieved and excluded articles at full text screening is shown in Additional file 4, together with a list of all included articles.

After the first round of double screening at the title and abstract level, when all reviewers were involved, Cohen’s kappa values of 0.52–0.63 were obtained in one group and 0.33–0.42 in the other group. In the second round, when only two reviewers were screening, the kappa value was 0.37 which is a relatively low value. However, after examining the disagreements, we found that most of them could be explained by the fact that one of the reviewers was too reluctant to exclude certain papers that quite clearly were not eligible (i.e., not mentioning gas fluxes in the title or abstract). Also, when the exclusion rate is very high (in our case > 95%), almost perfect agreement is required to obtain high kappa values. We concluded that it was safe to continue the screening in single mode. In the full text screening, Cohen’s kappa was 0.81.

**Fig. 2:**
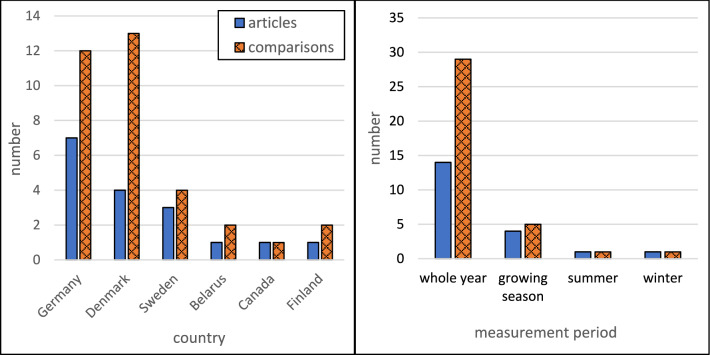
Geographical (left) and seasonal (right) distribution of articles or treatment comparisons included in the review. Only whole-year greenhouse gas measurements were included in the quantitative synthesis

**Fig. 3:**
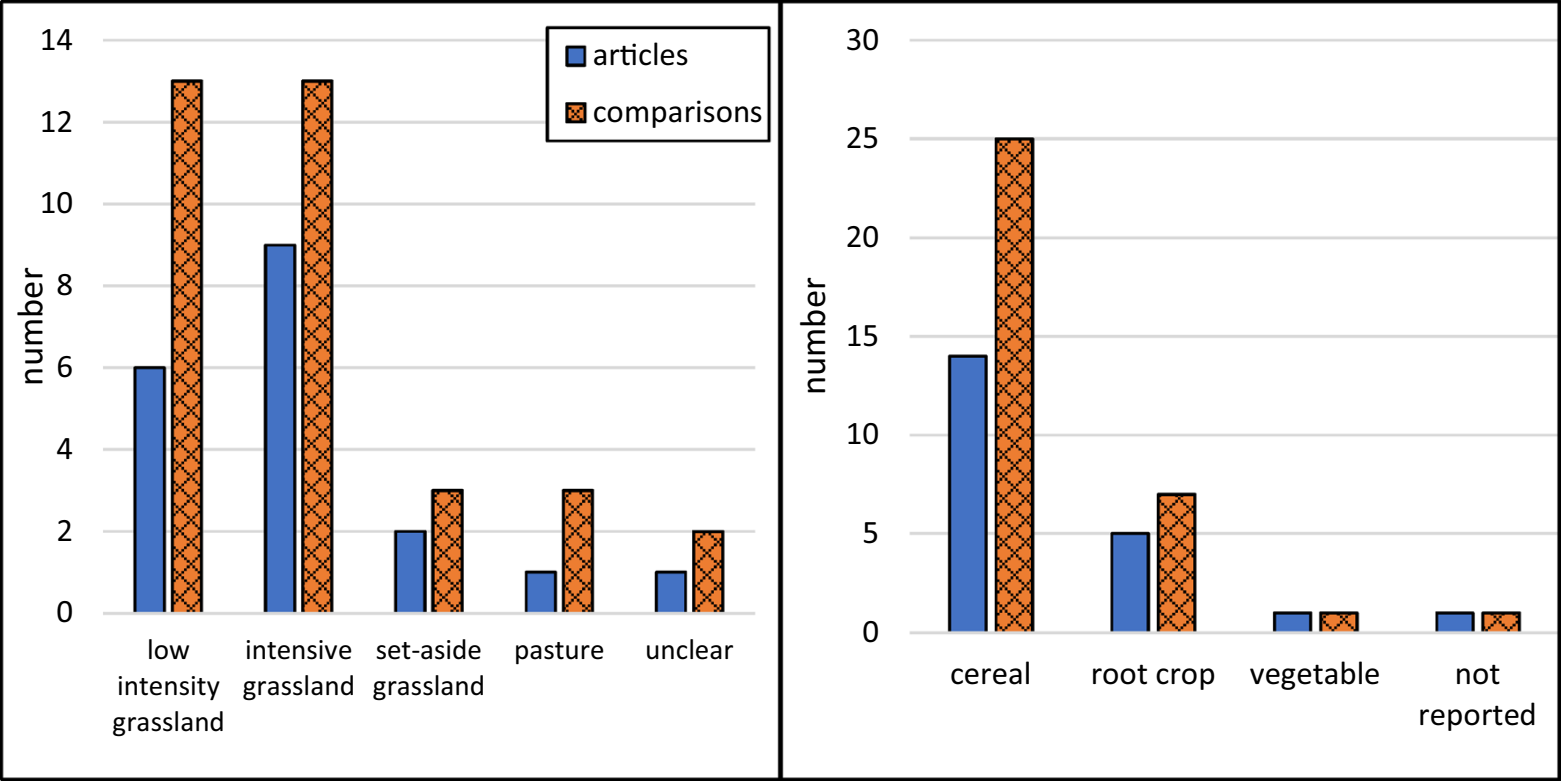
Distribution of grassland types (left) and crop types grown in croplands (right) in the articles or treatment comparisons within the selected articles

**Fig. 4:**
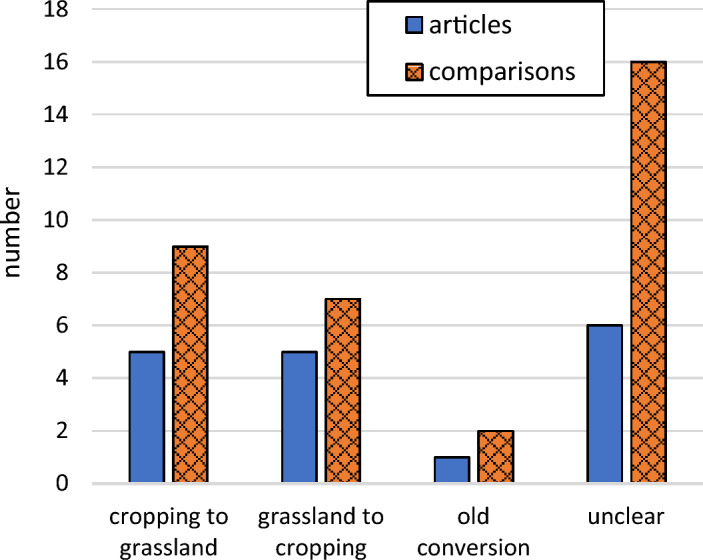
Distribution of land use conversions. The old conversion was at least 50 years old, meaning that the land use may be regarded as unchanged

**Fig. 5:**
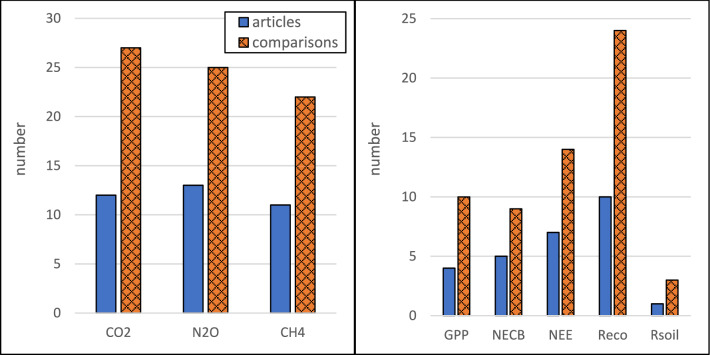
Left: number of articles and comparisons reporting fluxes of CO_2_, N_2_O, and CH_4_. Right: number of articles and comparisons reporting CO_2_ fluxes as Gross Primary Production (GPP), Net Ecosystem Carbon Balance (NECB), Net Ecosystem Exchange (NEE), ecosystem respiration (R_eco_), and soil respiration (R_soil_)

The included studies were performed in Germany [38–44], Denmark [37, 45–47], Sweden [20, 48, 49], Belarus [50], Finland [51], and Canada [52], and most studies reported whole-year measurements (Fig. 2). A clear majority of the studies were conducted in climate zone Cfb (22), followed by Dfb (4) and Dfc (2). In this systematic review, the studied permanent grasslands could be categorized as intensive grasslands (receiving N fertilization), low intensity grasslands (not receiving N fertilization), set-aside grasslands (not receiving N fertilization and not harvested), and pastures, while the croplands were categorized according to the dominating crop type as cereals, root crops, and vegetables (Fig. 3). All studies had a Control-Impact (CI) study design and most of them were preceded by some land use conversion. The land use history and land use conversion varied among the included studies, but the number of studies where land was converted from cropland to grassland was approximately equal to the number of studies where land was converted in the opposite direction (Fig. 4). Approximately the same number of studies reported fluxes of CO_2_, N_2_O, and CH_4_ (Fig. 5). Fourteen studies reported the fluxes of all three gases (CO_2_, N_2_O, and CH_4_). The most reported response variable for CO_2_ flux was R_eco_ followed by NEE (Fig. 5).

### Narrative synthesis including study validity assessment

Metadata and study validity assessments for all included studies are listed in a narrative synthesis table provided in Additional file 5. The table also indicates whether a specific comparison was used in the meta-analyses and, if not, the reason for not including it. Additional file 5 also includes a table with data used for meta-analyses.

**Fig. 6:**
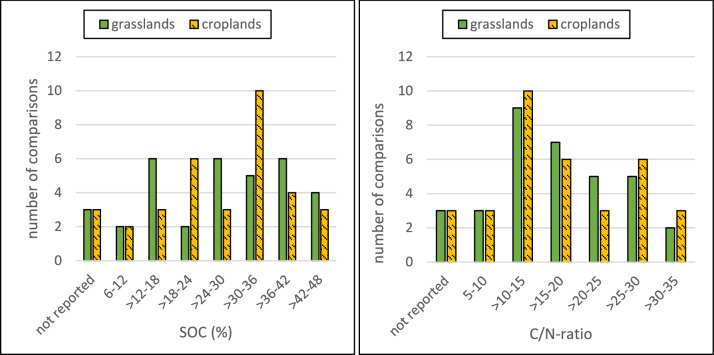
Distribution of soil organic carbon (SOC) concentration in the studied grasslands and croplands (left), and ratio of carbon to nitrogen (C/N) in  grassland and cropland (right)

**Fig. 7:**
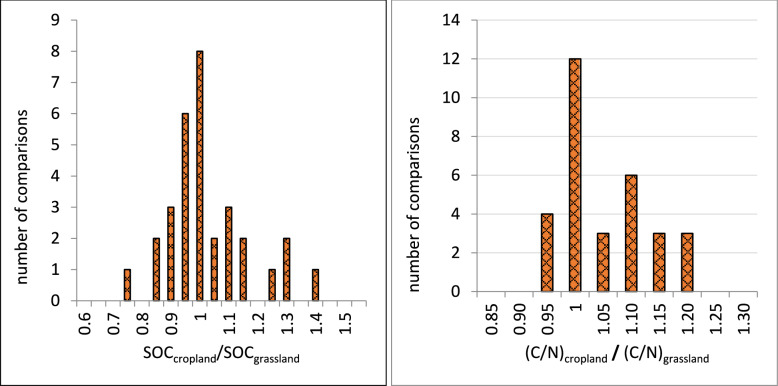
Ratios of soil properties between compared cropland and grassland, shown for SOC concentration (left) and soil C/N ratio (right). In most comparisons the ratio was close to 1, indicating that the studied soils were comparable in terms of SOC concentration and C/N ratio. Data not reported for three comparisons

**Fig. 8:**
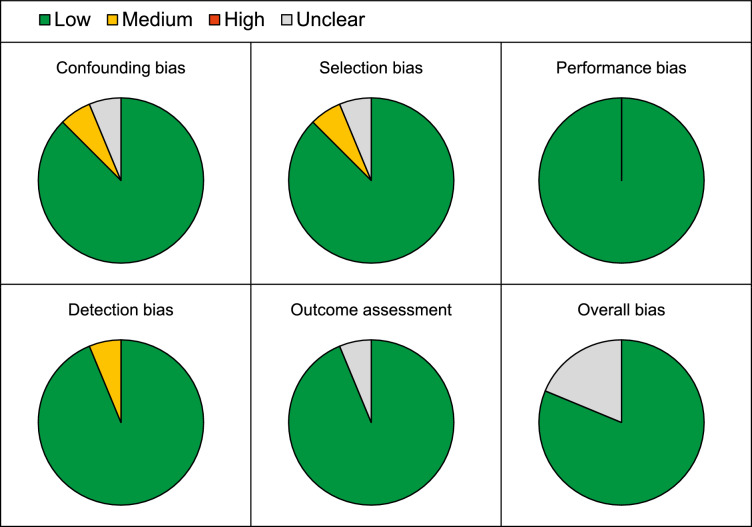
Risk of bias assessment at article level. The risk of bias was judged to be unclear and medium in three articles, resulting in unclear overall assessments (the risk of bias in those articles is at least medium, but may be even higher). In all other articles, the overall risk of bias was judged to be low

The SOC concentration is an important factor for the external validity of the studies. Based on our eligibility criteria, the SOC concentration is higher than 6% in all reported soils, and it is roughly evenly distributed between 12 and 42% (Fig. 6). Only two comparisons were found for soils with 6–12% SOC. Some articles did not report the SOC concentration but based on the reported soil taxa (Histosol) it was safe to assume that the soils in those articles were eligible for this review. The soil C/N ratio may also be an important factor regulating the GHG fluxes. In the included studies, the C/N ratio ranged between 9 and 35 (Fig. 6). For the internal validity of the studies, it is crucial that the SOC concentration and the C/N ratio is the same in both treatment groups. As shown in Fig. 7, the ratio of these parameters between the studied grasslands and croplands are generally close to 1, indicating that the studied soils are comparable in terms of SOC concentration and C/N ratio. Overall, the risk of bias in the included studies was judged to be low in all articles except three, where the risk of bias was assessed to be unclear (Fig. 8). In these three articles, however, the risk of at least one bias domain was judged to be medium.

### Data synthesis

**Fig. 9:**
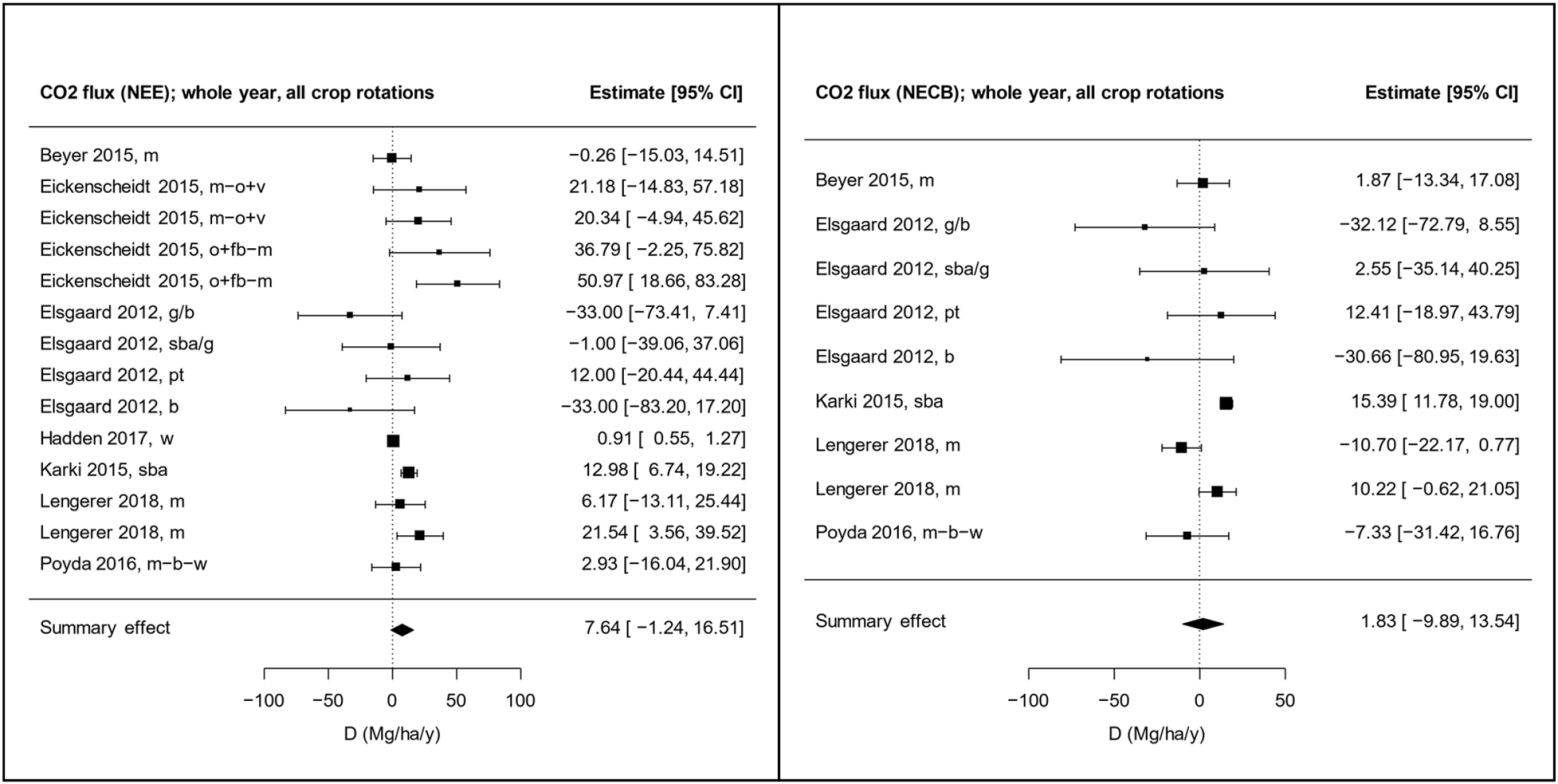
Forest plots showing raw mean differences in CO_2_ flux as Net Ecosystem Exchange (NEE) and Net Ecosystem Carbon Balance (NECB). The size of the squares is proportional to the weight of the studies, which is based on inverse variance. Letters after each study label indicate the crop rotation in the comparator group (see Additional file 5 for explanation). The raw mean difference, D, is the difference between fluxes from grassland and croplands (= grassland−cropland)

**Fig. 10:**
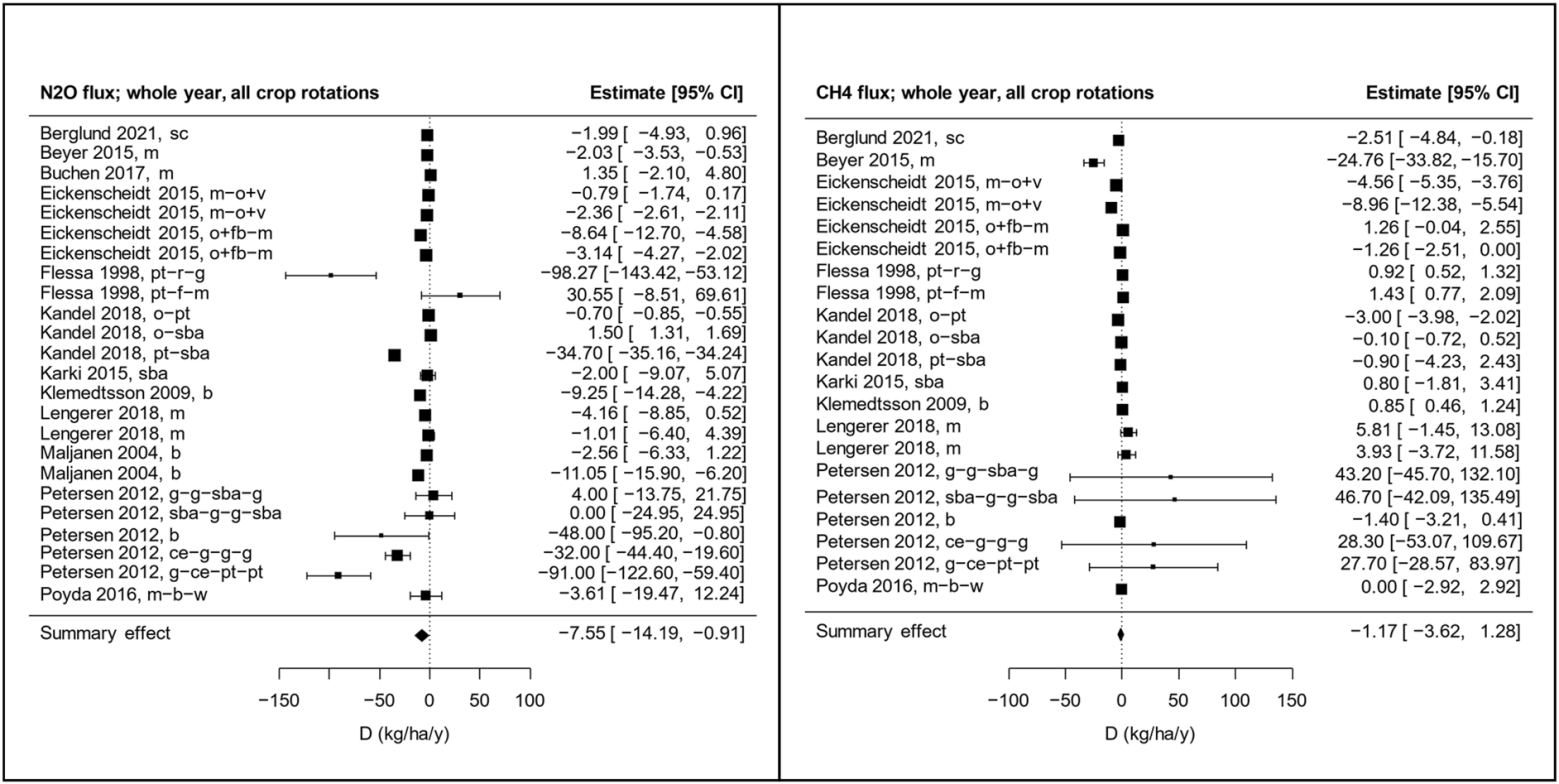
Forest plots showing raw mean differences in N_2_O flux (left) and CH_4_ (right). The size of the squares is proportional to the weight of the studies, which is based on inverse variance. Letters after each study label indicate the crop rotation in the comparator group (see Additional file 5 for explanation). The raw mean difference, D, is the difference between fluxes from grassland and croplands (= grassland−cropland)

Meta-analyses were conducted for NEE, NECB, R_eco_, N_2_O flux, and CH_4_ flux. Using an intercept-only model, the summary raw mean difference (D) was non-significant for all effect sizes of CO_2_ flux, i.e., D_NEE_, D_NECB_, and D_Reco_ (Fig. 9 and Additional file 6, Figure S1-S3). While D was non-significant also for the CH_4_ flux, it was significant for the N_2_O flux (Fig. 10). Including all types of crop rotations in the comparator group, the point estimate of the raw mean difference in N_2_O flux between grasslands and croplands (D_N2O_) was − 7.55 kg ha^−1^ y^−1^, with a 95% confidence interval of − 14.2 to − 0.91 kg ha^−1^ y^−1^. The negative numbers indicate that the N_2_O release was lower from grasslands than from croplands.

When including a single categorical moderator in the models, we found that the crop type grown on the croplands was a significant factor regulating the effect (p = 0.0007). When crop rotations involving root crops were excluded from the comparator group (i.e., only cereals were included), D_N2O_ was smaller but still significant, − 3.79 kg ha^−1^ y^−1^ with a 95% confidence interval of − 6.27 to − 1.31 kg ha^−1^ y^−1^ (Additional file 6**,** Figure S4). Thus, the difference in N_2_O release between grasslands and croplands tends to be greater when the croplands are used for root crop production. A regression model with comparator type as categorical moderator reveals that the subgroup with root crops results in a significantly more negative D_N2O_ than the subgroup with only cereals in the cropland (p = 0.0034, see Additional file 6**,** Table S2). We also tested the significance of climate zone, grassland type, and land use conversion as categorical moderators. Grassland type was a significant moderator for NEE and NECB. Comparing the subgroups, both D_NEE_ and D_NECB_ were significantly higher for intensive grasslands than for low intensity grasslands. This means that grasslands receiving N fertilization lost more carbon than the non-fertilized grasslands in relation to the croplands.

**Fig. 11:**
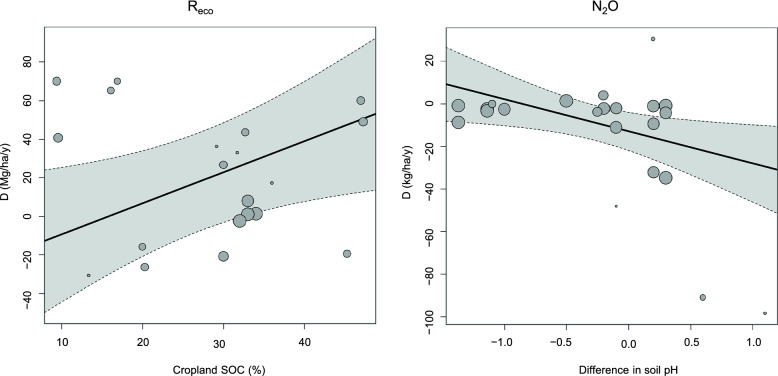
Regression plots showing significant correlations between D_Reco_ and cropland SOC (left), and between D_N2O_ and difference in soil pH between grasslands and croplands (right). All crop rotations are included in the comparator group

Meta-regressions were also conducted where single continuous variables were used as moderators. The results of the meta-regressions are shown in Additional file 6, Table S3. When including all types of crop rotations in the comparator group, we found the statistically significant correlations shown in Table 2. Regression plots for those correlations are shown in Additional file 6, Figure S6–S10. For D_NEE_, we found positive correlations with grassland N fertilization, the difference in N fertilization between grassland and cropland (ΔN fertilization), and average N fertilization. The first two correlations indicate that the more the grasslands are fertilized, the more positive becomes the difference in NEE between grasslands and croplands (i.e., more CO_2_ is released from the grassland compared to the cropland). This effect of grassland fertilization on NEE is in line with findings in other studies [53–55]. There is a positive correlation between D_NECB_ and grassland SOC concentration, indicating that high grassland SOC concentrations result in higher C export from grasslands compared to croplands. A positive correlation was also seen between D_Reco_ and cropland SOC concentration (Fig. 11). We also found a positive correlation between D_N2O_ and cropland soil pH and a negative correlation between D_N2O_ and ΔpH, indicating that increasing cropland soil pH results in lower N_2_O emissions from croplands compared to grasslands. This makes sense since low pH limits the activity of the enzyme N_2_O reductase and thus increases the ratio of the end products N_2_O and N_2_ (N_2_O/(N_2_O + N_2_) ratio) in denitrification [56–58]. It should be noted here that in an ideal study the management and soil properties of the grassland plots should be identical to those of the cropland plots. A correlation between an effect size and a difference in management or a soil property between the treatment groups may indicate that the summary effect obtained using an intercept-only model could be biased (baseline confounding or post-intervention selection bias). For example, the observed significant summary effect on N_2_O flux in our meta-analysis could potentially be biased by systematic differences in soil pH between grasslands and croplands. However, looking at Fig. 11, we see that both positive and negative differences in pH are included, and that at ΔpH = 0, D_N2O_ is negative (− 12.7 kg ha^−1^ y^−1^, p = 0.0075), meaning that the N_2_O flux is greater from cropland than from grassland at similar pH. It is thus unlikely that the significant summary effect on the N_2_O flux obtained by the intercept-only model (Fig. 10) is caused by systematic differences in soil pH. We also note that the intercept according to the regression model (Fig. 11) is within the 95% confidence interval obtained by the intercept-only model (Fig. 10).

**Table 2:** Significant correlations found in meta-regressions using models with a single continuous moderator (k is the number of comparisons used in the analysis)

Effect size	Moderator	Coefficient	p-value	k
D_N2O_	Cropland soil pH	9.18874	0.0238	24
D_N2O_	Difference in soil pH	− 15.03957	0.02278	22
D_NECB_	Grassland SOC (%)	1.59462	0.0307	9
D_NEE_	Grassland soil C/N ratio	− 2.01589	0.00865	14
D_NEE_	Grassland N fertilization (kg/ha/y)	0.20856	0.00044	13
D_NEE_	Difference in N fertilization (kg/ha/y)	0.26556	0.04238	9
D_NEE_	Average soil C/N ratio	− 1.60731	0.03881	14
D_NEE_	Average N fertilization (kg/ha/y)	0.1647	0.04677	13
D_Reco_	Cropland SOC (%)	1.60996	0.02512	20
D_Reco_	Difference in soil C/N ratio	− 7.29392	0.04611	19
D_Reco_	Difference in soil pH	17.44507	0.04854	19

A complete list of tested correlations can be found in Additional file 6, Table S3

The *I*^*2*^ statistic was used to assess the heterogeneity among comparisons (see Table S1 in Additional file 6). The total heterogeneity is in general relatively high (> 60% of the observed variation between comparisons could be explained by true differences). However, in line with the recommendations by Nakagawa et al. [33], we also examined the heterogeneity among the included studies, or more precisely among the study locations in our case. It was then revealed that the heterogeneity in effect on N_2_O flux was rather low (14% when all crop rotations are included and close to 0% when root crops are excluded). The heterogeneity among study locations was close to 0% also for NECB. Accordingly, it is reasonable to assume that converting a certain cropland on organic soils to a certain grassland will have approximately the same effect on N_2_O emissions wherever it is implemented within the Cfb, Dfa, Dfb, and Dfc climate zones.

#### Risk of bias and robustness

The overall risk of bias in the studies was judged to be unclear in three of the included articles. When only studies with low risk of bias were included in the analyses, most of the results above remained (see Additional file 6, Table S4–S6). For example, D_N2O_ was still significant (p = 0.0402). However, a leave-one-out analysis (Additional file 6, Table S7) showed that omitting the paper by Maljanen et al. [51] resulted in a non-significant D_N2O_ (p > 0.05), indicating that the significant result is not robust. Moreover, funnel plots and Egger’s regression tests (Additional file 6, Figure S11), indicate that the risk of publication bias should not be ignored for N_2_O measurements. Considering these findings, we cannot with certainty say that converting cropland to grassland has a significant effect on N_2_O emissions from organic soils.

## Review limitations

### Limitations of the review methods

Our search for both peer-reviewed and grey literature was comprehensive, but it is possible that some relevant studies were not identified. The search was limited to publications written in English, Swedish, Finnish, French, Danish and German language, and some studies published in other languages may have been excluded. Additionally, even though we could find numerous relevant grey literature reports, some eligible reports might not have been captured, potentially introducing publication bias into our review. However, as measurements of annual GHG fluxes from organic soils are costly and labor intensive, we expect most of the completed studies to be published in scientific journals so that the effort is recognized. Despite an extensive search, there were papers that we were unable to locate or access. We applied strict inclusion and exclusion criteria to ensure the eligibility of the studies included in the review. Consequently, the small number of included studies prevented us from investigating interactions between different effect moderators. For instance, 13 papers with grassland sites that had been established for less than 3 years (see Additional file 4) were excluded for the present analysis but could possibly provide additional insights.

### Limitations of the evidence base

Although our eligibility criteria aimed at including only studies with comparable fields within each study, some included studies were assessed as having an unclear but at least medium risk of bias. These biases were due to factors such as unclear distance between comparisons, unusual crop treatment (e.g., lower N fertilization in cereals), differences in water or fertilization regimes, or slightly different soil properties between treatment groups. Such differences in baseline conditions between studies can confound the results, making it challenging to draw definitive conclusions about the effectiveness of the treatments.

The meta-data of the studies included in this review were in some cases poorly reported. Notably, there was a lack of consistency in the reporting of key variables such as total N, C/N ratio or phosphorus concentration, but also, e.g., time since drainage and tillage intensities on croplands were rarely reported. Missing data on the start of the intervention (time of establishment of the grassland) were a reason for excluding articles, if no further information could be obtained by contacting the corresponding author of the study in question. Such gaps in information hinder the ability to perform a comprehensive meta-analysis and reduce the generalizability of the findings. Furthermore, many studies did not provide detailed methodological descriptions, which limited our ability to fully assess their validity.

## Review conclusions

### Implications for policy/management

There was no statistically significant difference in CO_2_ emissions between croplands and grasslands whether calculated as R_eco_, NEE or NECB (Fig. 9 and Additional file 6). This implies that merely a change from an annual crop to grassland does not lead to significant CO_2_ emission reduction if site conditions are not changed to allow for reduced peat decomposition. This aligns with the results from a recent study by Keck et al. [59], that was published after the search for articles was completed for this present systematic review. The IPCC EFs suggest a difference of 6.6 to 16 Mg CO_2_ ha^−1^ y^−1^ between croplands and grasslands (depending on the grassland type) in the temperate region [22]. Based on our results, this results likely more from different site characteristics (e.g., soil properties and management) in grasslands than the cropping type itself.

Methane fluxes were also unaffected by the land use when the comparison was done in similar site conditions (Fig. 10). This differs from the earlier analysis done to develop EFs for GHG reporting [22]. The IPCC default emission factors for the temperate region assume an increase of CH_4_ emissions from zero in cropland to 1.8‒39 kg ha^−1^ y^−1^ in grassland depending on the nutrient and drainage status of the field. It is likely that grassland sites are generally wetter and emit more CH_4_ than cropland sites, but our results suggest that a land use change from cropland to grassland does not increase CH_4_ emissions in well-drained sites.

N_2_O fluxes were reduced by an average of 7.55 kg ha^−1^ y^−1^ in grassland compared to cropland (Fig. 10). This estimate is similar to the difference in IPCC emission factors between cropland and deep drained nutrient rich grassland in the temperate region of 7.54 kg ha^−1^ y^−1^ [22]. The mitigation effect is not negligible as it corresponds to about 2 Mg of CO_2_ equivalent emissions and thus exceeds the effect of most carbon farming measures on mineral soils [60]. However, there are a couple of large reduction rates among the observations in our comparison that increase the average reduction rate [42, 47] and we cannot with certainty say that converting cropland to grassland has a significant effect on N_2_O emissions from organic soils. The main reason for the lower emissions with perennials is likely the longer period for nutrient uptake by grass as compared to annual crops, leaving less mineral nitrogen available for nitrifying and denitrifying microbes. Higher soil nitrate concentration and positive correlation to N_2_O fluxes have been observed, e.g., in a study comparing grass and cereal production in peat [61] and sandy soil [62]. Particularly high emissions of N_2_O have been reported for row cropping of potatoes [46, 47].

It would be important to abate especially CO_2_ emissions that represent a major part of the climate impact of cultivated peat soils. As CO_2_ emissions were not reduced according to our results, it can be expected that conversion of an arable peatland to grassland does not lead to large benefits in terms of climate change mitigation. It is thus crucial to implement more cost-effective measures to abate GHG emissions from organic soils in the agri-environmental schemes.

The results also point to the possibility that liming reduces N_2_O emissions in peat soils, as has been found for mineral soils in laboratory [56] and field studies [63]. The mechanism behind this is related to low pH inhibiting the enzyme converting N_2_O to N_2_ [56]. However, lime itself releases CO_2_ which partly counteracts the potential benefit in GHG mitigation [64].

### Implications for research

The evidence base of this systematic review was narrow, as many papers on the topic had limitations that prevented their inclusion. Therefore, this section will give some recommendations on what factors to look out for when designing future research.

A major limitation identified in many of the papers in the systematic review is the geographical dispersion of the field trials and treatments, coupled with a limited number of measurements and the use of pseudo-replicates rather than true replicates. To improve comparability between treatments and to reduce potential gradients in timing of measurement or soil characteristics, it is recommended to use a randomized block design. If this is not feasible, it could be relevant, e.g., to place different treatments in adjacent fields at equal distances from ditches, rather than spreading the sites over a larger area. This approach helps maintain consistency and reduce variability in experimental conditions.

It is challenging to estimate the proportion of CO_2_ emissions originating from peat decomposition which should be the primary basis of CO_2_ emission factors used in the greenhouse gas reporting [22]. Peat decomposition can be estimated based on soil respiration measurements on bare soil, but the loss of peat is likely to be underestimated in the absence of input of fresh carbon in crop residues and root exudates, which likely prime peat decomposition [65]. Net ecosystem exchange is also an incomplete estimate of the carbon balance as it excludes the amount of carbon exported in the harvest yields and includes plant respiration in addition to soil respiration. Thus, in the case of studies based on the balance of carbon losses and gains, the NEE should be supplemented with the estimate of carbon exported at harvest to obtain the NECB. In the reviewed papers, full carbon balances or yield data were rarely available, and this adds uncertainty to the conclusions.

Because soil processes evolve rapidly on drained organic soils, it is imperative to characterize them thoroughly when conducting GHG flux studies [66]. Different types of drained organic soils exhibit different behaviors, particularly in terms of compaction, water movement, and nutrient cycling. Therefore, studies comparing treatments (e.g., cropping systems, amendments, fertilization) must ensure that similar soils are compared, which requires proper characterization. Essential soil parameters to be included in studies encompass primarily chemical and physical properties that provide insight into the degradation state of the peat soil. These parameters include total C and N, indicative of the C/N ratio, degree of decomposition, peat thickness, pH, and bulk density. Additionally, information such as cropping history, drainage date and depth, and water table depth are crucial as they can significantly impact soil processes in drained organic soils [66].

The observation that CO_2_ emissions from organic soils are not directly dependent on land use in paired studies (cropland versus grassland) means that other soil factors should be identified, which can explain the difference in CO_2_ emissions among organic soils. Single chemical properties are generally insufficient proxies for CO_2_ emissions [67], but several studies have identified water table depth as an overarching driver of CO_2_ emissions under field conditions, as higher water tables reduce CO_2_ emissions by limiting O_2_ availability [9, 68, 69]. Recent studies show that SOC concentration and SOC density as such may not be strong predictors of CO_2_ emissions [13] although SOC is the direct source for heterotrophic CO_2_ production. This lack of causality may at least partly be explained by the fact that the SOC comprises different fractions of carbon compounds with varying microbial availability and intrinsic decomposability, which influences the rate of CO_2_ emission [70]. Chemical or physico-chemical characterization of SOC pools in peat soils may have a potential to inform on decomposability which could be explored in analyses of CO_2_ emission. For example, Petersen et al. [71] employed Extended Slow Heating (ESH) pyrolysis for characterizing the microbial availability of different carbon fractions in biochar. This technique could be applied to peat soils, offering new insights into their carbon composition and microbial accessibility. Recent studies have also found that other soil parameters might be important in distinguishing organic soil types and explaining the GHG emissions. These parameters include total phenolic compounds [72, 73], total microbial activity as measured by fluorescein diacetate hydrolysis [72] and the concentration of elements such as iron, sulphur and phosphorus [74–76].

In summary, the GHG emissions in cultivated peatlands should be estimated by methods that take into account more variables than simply the cultivated crop. The overriding effect of water table depth is already well known (e.g., [9]) and data on that can be used as a basis of the national estimates if measurements or modelled estimates are available. In addition to the effect of peat physico-chemical properties, the role of SOC quality and microbial community structure and functions need to be addressed.

## Supplementary Information

Additional file 1: Search strategy and search results.

Additional file 2: Data extraction templates.

Additional file 3: R code for meta-analysis.

Additional file 4: Decisions at full text screening.

Additional file 5: Narrative synthesis tables.

Additional file 6: Meta-analysis results.

Additional file 7: ROSES for Systematic Review Reports.

## Data Availability

All data generated or analysed during this study are included in this published article and its supplementary information files. The datafile and R code for replicating the analyses and figures are archived at Zenodo (10.5281/zenodo.14217560).
