# Supplementary material for: Effects of converting cropland to grassland on greenhouse gas emissions from peat and organic-rich soils in temperate and boreal climates: a systematic review
Source: Environ Evid. 2025 Jan 19;14:1. doi: 10.1186/s13750-024-00354-1 (PMC11743012; doi:10.1186/s13750-024-00354-1)
Supplement: Supplementary file 1 — Additional file 1: Search strategy and search results. [file 13750_2024_354_MOESM1_ESM.docx]

# Search strategy and search results

**Table 1.** Bibliographic databases used for searching.

| **Database/platform** | **Publisher and URL** |
| --- | --- |
| Web of Science^1)^ | Clarivate Analytics, <https://clarivate.com/products/web-of-science/> |
| Scopus | Elsevier, <https://www.scopus.com/> |
| ProQuest Natural Science Collection^2)^ | Proquest, <https://www.proquest.com/> |
| CAB Abstracts^3)^ | Clarivate Analytics, <https://clarivate.com/products/web-of-science/> |
| Directory of Open Access Journals | Independent, <https://doaj.org/> |

^1)^ Web of Science^TM^ Core Collection, including: Science Citation Index Expanded (SCI-EXPANDED), Social Sciences Citation Index (SSCI), Arts & Humanities Citation Index (A&HCI), Conference Proceedings Citation Index- Science (CPCI-S), Conference Proceedings Citation Index- Social Science & Humanities (CPCI-SSH) and Emerging Sources Citation Index (ESCI).

^2)^ Including AGRICOLA, Agricultural Science database, Environmental Science database, Environmental Science index, Biological Science database, Biological Science index, Earth, atmosphere & Aquatic Science database.

^3)^ Subscription through Clarivate.

**Table 2.** Search strings in individual bibliographic databases.

| **Database** | **Search string** |
| --- | --- |
| Web of Science | **(**“organic soil” OR “organic soils” OR peatland OR peatlands OR histosol* OR “muck sediment” OR “muck sediments” OR “muck soil” OR ”muck soils” OR gyttja OR moorsh* OR wetland* OR turf* OR coprogenous OR muskeg OR suo OR mud OR muds OR swamp OR swamps OR lowland* OR fen* OR mire OR mires OR marsh* OR morass OR quag* OR gley* OR “carbon rich” OR “black soil” OR “black soils” OR bog* OR “high organic carbon” OR hydromorphic* **) AND (**grass OR grassland* OR ley* OR fallow OR pasture OR forage OR perennial* OR grazing OR mesocosm* OR lysimeter* OR semifield* OR legume* OR pulse* OR alfalfa* OR lupin* OR bean* OR lentil* OR clover* OR meadow* OR timothy OR set-aside OR setaside OR “pea” OR “peas”**) AND (**greenhouse gas OR “greenhouse gases” OR “carbon dioxide” CO2* OR “carbon emission” OR “carbon emissions” OR “nitrous oxide” OR “nitrous oxides” OR N20 OR “laughing gas” OR methane OR CH4 OR “global warming potential” OR ghg* OR “net ecosystem exchange” OR “net ecosystem production” OR respiration OR “carbon balance” OR “trace gas” OR “trace gases” OR nee OR nep OR “carbon turnover” OR “eddy covariance”**)** |
| Scopus | **(**{organic soil} OR {organic soils} OR peatland OR peatlands OR histosol* OR {muck sediment} OR {muck sediments} OR {muck soil} OR {muck soils} OR gyttja OR moorsh* OR wetland* OR turf* OR coprogenous OR muskeg OR suo OR mud OR muds OR swamp OR swamps OR lowland* OR fen* OR mire OR mires OR marsh* OR morass OR quag* OR gley* OR {carbon rich} OR {black soil} OR {black soils} OR bog* OR {high organic carbon} OR hydromorphic***) AND (**grass OR grassland* OR ley* OR fallow OR pasture OR forage OR perennial* OR grazing OR mesocosm* OR lysimeter* OR semifield* OR legume* OR pulse* OR alfalfa* OR lupin* OR bean* OR lentil* OR clover* OR meadow* OR timothy OR set-aside OR setaside OR {pea} OR {peas}**) AND (**{greenhouse gas} OR {greenhouse gases} OR {carbon dioxide} CO2* OR {carbon emission} OR {carbon emissions} OR {nitrous oxide} OR {nitrous oxides} OR N20 OR {laughing gas} OR methane OR CH4 OR {global warming potential} OR ghg* OR {net ecosystem exchange} OR {net ecosystem production} OR respiration OR {carbon balance} OR {trace gas} OR {trace gases} OR nee OR nep OR {carbon turnover} OR {eddy covariance}**)** |
| CAB Abstracts | Same as Web of Science |
| Proquest | Same as Web of Science |
| DOAJ^1)^ | https://doaj.org/api/v3/search/articles/("organic soil" OR "organic soils" OR peat OR histosol OR "muck sediment" OR "muck sediments" OR "muck soil" OR "muck soils" OR gyttja OR moorsh OR wetland OR turf OR coprogenous OR muskeg OR suo OR mud OR muds OR swamp OR swamps OR lowland OR fen OR fens OR mire OR mires OR marsh OR morass OR quag OR gley OR "carbon rich" OR "black soil" OR "black soils" OR bog OR "high organic carbon" OR hydromorphic) **AND** (grass OR grassland OR ley OR fallow OR pasture OR forage OR perennial OR mesocosm OR lysimeter OR semifield OR legume OR pulse OR alfalfa OR lupin OR bean OR lentil OR clover OR meadow OR timothy OR set-aside OR setaside OR pea OR peas OR crop OR graz) **AND** ("greenhouse gas" OR "greenhouse gases" OR "carbon dioxide" OR CO2 OR "carbon emission" OR "carbon emissions" OR "nitrous oxide" OR "nitrous oxides" OR N2O OR "laughing gas" OR methane OR CH4 OR "global warming potential" OR GHG OR "net ecosystem exchange" OR "net ecosystem production" OR respiration OR "carbon balance" OR "trace gas" OR "trace gases" OR NEE OR NEP OR "carbon turnover" OR "eddy covariance" OR "dinitrogen oxide" OR "dinitrogen monoxide" OR "marsh gas") |

^1)^ Wildcards are not allowed. The search was conducted through an Application Programming Interface (API) request rather than the search web page.

**Table 3.** Search results in bibliographic databases.

| **Database/platform** | **Date** | **Searched field** | **Records** | **Records safter deduplication** |
| --- | --- | --- | --- | --- |
| Web of Science^1)^ | 2023-01-20 | topic | 4012 | 4008 |
| CAB Abstracts | 2023-01-20 | topic | 5578 | 3384 |
| Scopus | 2023-01-20 | title, abstract and keywords | 3783 | 926 |
| ProQuest Natural Science Collection^2)^ | 2023-01-20 | abstract | 3013 | 576 |
| Directory of Open Access Journals | 2023-01-20 | all fields | 330 | 330 |

^1)^ Web of Science^TM^ Core Collection.

^2)^ Including AGRICOLA, Agricultural Science database, Environmental Science database, Environmental Science index, Biological Science database, Biological Science index, Earth, atmosphere & Aquatic Science database.

**Table 4.** Searches in BASE (Bielefeld Academic Search Engine, <https://www.base-search.net/>). The first 300 records (sorted on relevance) found using search words in English were screened.

| **Language** | **Searched field** | **Search string** | **Date** | **Records** |
| --- | --- | --- | --- | --- |
| English | title | cultivated arable crop* grass*) AND (peat histosol "organic soil") AND (“greenhouse gas” “greenhouse gases” “carbon dioxide” CO2 “nitrous oxide” N2O methane CH4) | 2024-03-14 | 2273 |
| French | entire document | ("tourbière agricole" "tourbière cultivée" "sol organique" histosol) AND (respiration "gaz à effet de serre" "dioxide de carbone" méthane "oxyde nitreux" "protoxyde d'azote") | 2024-03-13 | 123 |
| German | entire document | moor AND (kohlenstoff* treibhausgas* CO2 N2O CH4) | 2024-03-21 | 88 |
| Danish | entire document | (Jord* dyrket* mark* omdrift* græs*) AND (organisk* lavbund* tørv* kulstofrig*) | 2024-02-27 | 202 |

**Table 5.** Searches in Finna (<https://finna.fi/?lng=en-gb>).

| **Language** | **Searched field** | **Search string** | **Date** | **Records** |
| --- | --- | --- | --- | --- |
| English | all fields | (cultivated OR arable OR crop* OR grass*) AND (peat OR histosol OR "organic soil") AND ("greenhouse gas" OR "greenhouse gases" OR "carbon dioxide" OR CO2 OR "nitrous oxide" OR N2O OR methane OR CH4) | 2024-03-15 | 77 |
| Finnish | all fields | (turvemaa OR orgaaninen maa OR eloperäinen maa OR lieju OR turvepelto OR eloperäinen peltomaa) AND (maankäyttö OR Nurmi OR vilja) AND (kasvihuonekaasu OR hiilidioksidi OR metaani OR dityppioksidi OR ilokaasu) | 2024-03-03 | 209 |
| Finnish | all fields | (turvemaa OR orgaaninen maa OR eloperäinen maa OR lieju OR turvepelto OR eloperäinen peltomaa) AND (kasvihuonekaasu OR hiilidioksidi OR metaani OR dityppioksidi OR ilokaasu) | 2024-03-03 | 307 |

**Table 6.** Searches in Swepub (<https://swepub.kb.se/>).

| **Language** | **Searched field** | **Search string** | **Date** | **Records** |
| --- | --- | --- | --- | --- |
| English | entire document | (cultivated OR arable OR crop* OR grass*) AND (peat OR histosol OR "organic soil") AND ("greenhouse gas" OR "greenhouse gases" OR "carbon dioxide" OR CO2 OR "nitrous oxide" OR N2O OR methane OR CH4) | 2024-03-15 | 66 |
| Swedish | entire document | (torv OR kärrtorv OR mosstorv OR mulljord OR mull OR gyttja OR gyttjejord OR myr OR svartjord OR organogen jord OR bleke OR kalkgyttja OR dy OR svartmocka) AND (vall OR bete OR betesvall OR slåttervall OR naturbete OR träda OR svartträda OR övergiven OR öppen odling OR stråsäd OR oljeväxter OR spannmålsodling OR rotfrukter,potatis OR grönsaksodling OR energiskogsodling) AND (koldioxid OR lustgas OR metan OR dikväveoxid OR växthusgaser) | 2024-03-15 | 3 |

**Table 7.** Searches in ProQuest Dissertations and Theses (<https://www.proquest.com/>).

| **Language** | **Searched field** | **Search string** | **Date** | **Records** |
| --- | --- | --- | --- | --- |
| English | Abstract and summary text | (cultivated OR arable OR crop* OR grass*) AND (peat OR histosol OR "organic soil") AND ("greenhouse gas" OR "greenhouse gases" OR "carbon dioxide" OR CO2 OR "nitrous oxide" OR N2O OR methane OR CH4) | 2024-03-15 | 3 |

**Table 8.** Search strings used for searches in Google Scholar. The first 300 records retrieved by each search string were combined and then deduplicated, resulting in 1081 unique records. Terms in red text indicate a difference compared to the search string in the line above. Searches were performed 2023-01-27 and restricted to PDF documents.

| **No.** | **Search string** |
| --- | --- |
| 1 | cultivated **AND** peat **AND** **(**“greenhouse gas” OR “greenhouse gases” OR “carbon dioxide” OR CO2 OR “nitrous oxide” OR N2O OR methane OR CH4**)** |
| 2 | arable **AND** peat **AND** **(**“greenhouse gas” OR “greenhouse gases” OR “carbon dioxide” OR CO2 OR “nitrous oxide” OR N2O OR methane OR CH4**)** |
| 3 | crop **AND** peat **AND** **(**“greenhouse gas” OR “greenhouse gases” OR “carbon dioxide” OR CO2 OR “nitrous oxide” OR N2O OR methane OR CH4**)** |
| 4 | cultivated **AND** “organic soil” **AND (**“greenhouse gas” OR “greenhouse gases” OR “carbon dioxide” OR CO2 OR “nitrous oxide” OR N2O OR methane OR CH4**)** |
| 5 | arable **AND** “organic soil” **AND (**“greenhouse gas” OR “greenhouse gases” OR “carbon dioxide” OR CO2 OR “nitrous oxide” OR N2O OR methane OR CH4**)** |
| 6 | crop **AND** “organic soil” **AND (**“greenhouse gas” OR “greenhouse gases” OR “carbon dioxide” OR CO2 OR “nitrous oxide” OR N2O OR methane OR CH4**)** |
