# Supplementary material for: Effects of converting cropland to grassland on greenhouse gas emissions from peat and organic-rich soils in temperate and boreal climates: a systematic review
Source: Environ Evid. 2025 Jan 19;14:1. doi: 10.1186/s13750-024-00354-1 (PMC11743012; doi:10.1186/s13750-024-00354-1)
Supplement: Supplementary file 3 — Additional file 3: R code for meta-analysis. [file 13750_2024_354_MOESM3_ESM.docx]

# R code for meta-analysis

In the following R code, dataX is a data frame containing a selected subset of data read from the data table in Additional file 5 (e.g., whole-year data for NEE).

**## Start of code ##**

library(metafor)

**## Construct a variance-covariance matrix ##**

## The variance covariance is due to reuse of information from a ‘shared’ condition (one intervention ## and multiple controls) in some of the studies. The sample sizes (weights) are equal across
## conditions in all comparisons, which implies a correlation of 0.5.

VCV <- vcalc(vi = (dataX$se)^2, # sampling variances that are correlated within the same study;
cluster = dataX$Study.ID, # study identity - clustering variable;
grp1 = dataX$INTERVENTION, grp2 = dataX$crop.rotation,
w1 = datar$n_int, w2 = datar$n_comp # use sample size as weight
)

**## Perform analysis accounting for dependencies among both effect sizes and sampling errors ##**

## In the intercept-only model the mods argument is omitted.

flux <- rma.mv(yi = D,
 V = VCV,
 mods = ~ moderator, # “moderator” is an optional moderator.
 random = list(~1 | location,
 ~1 | comparison.ID),
 method = "REML",
 data = dataX,
 test = "t"
 )

**## Calculate total heterogeneity and heterogeneity among study locations ##**

library(orchaRd) # OrchaRd installation instructions from <https://github.com/daniel1noble/orchaRd>

# (https://besjournals.onlinelibrary.wiley.com/doi/full/10.1111/2041-210X.14152)

I2tot <- i2_ml(flux)[1]
I2loc <- i2_ml(flux)[2]

**## Construct a funnel plot and perform Egger’s regression test ##**

funnel(flux)

# The regtest function in the metafor package does not work with objects of class “rma.mv”. If effect # sizes and standard errors are passed as vectors to the function via the arguments x and sei, regtest # by default uses a mixed-effects meta-regression model for the regression test. However, as a
# multilevel meta‑analytical model was used in this review, we perform the test using the rma.mv
# function with standard error as moderator.

rma.mv(yi = D,
 V = VCV,
 mods = ~ se,
 random = list(~1 | location,
 ~1 | comparison.ID),
 method = "REML",
 data = dataX,
 test = "t"
 )
