# Supplementary material for: Effects of converting cropland to grassland on greenhouse gas emissions from peat and organic-rich soils in temperate and boreal climates: a systematic review
Source: Environ Evid. 2025 Jan 19;14:1. doi: 10.1186/s13750-024-00354-1 (PMC11743012; doi:10.1186/s13750-024-00354-1)
Supplement: Supplementary file 6 — Additional file 6: Meta-analysis results. [file 13750_2024_354_MOESM6_ESM.docx]

# Meta-analysis results

## Intercept-only model

**Table S1.** Compilation of results from meta-analyses using intercept-only models. D is raw mean difference (=X_grassland_ – X_cropland_ where X is mean flux), k is number of comparisons, and I^2^_tot_ and I^2^_loc_ is total heterogeneity and heterogeneity among study locations, respectively.

| **Comparator** | **Response** | **Summary D [95% CI]** | **p-value** | **k** | **I^2^_tot_ (%)** | **I^2^_loc_ (%)** |
| --- | --- | --- | --- | --- | --- | --- |
| all crop rotations | NEE (Mg/ha/y) | 7.64 [-1.24, 16.5] | 0.0858 | 14 | 68 | 68 |
| all crop rotations | Reco (Mg/ha/y) | 18.2 [-6.32, 42.7] | 0.137 | 20 | 100 | 78 |
| all crop rotations | NECB (Mg/ha/y) | 1.83 [-9.89, 13.5] | 0.728 | 9 | 70 | 0.00000057 |
| all crop rotations | N2O (kg/ha/y) | -7.55 [-14.2, -0.912] | **0.0276** | 24 | 100 | 14 |
| all crop rotations | CH4 (kg/ha/y) | -1.17 [-3.62, 1.28] | 0.33 | 21 | 98 | 32 |
| no root crop | NEE (Mg/ha/y) | 7.33 [-2.26, 16.9] | 0.122 | 13 | 72 | 72 |
| no root crop | Reco (Mg/ha/y) | 15 [-9.27, 39.3] | 0.207 | 16 | 97 | 86 |
| no root crop | NECB (Mg/ha/y) | 0.697 [-12.4, 13.8] | 0.903 | 8 | 74 | 0.00000037 |
| no root crop | N2O (kg/ha/y) | -3.79 [-6.27, -1.31] | **0.0048** | 19 | 99 | 0.00000021 |
| no root crop | CH4 (kg/ha/y) | -1.79 [-5.96, 2.37] | 0.374 | 16 | 99 | 55 |

### Forest plots


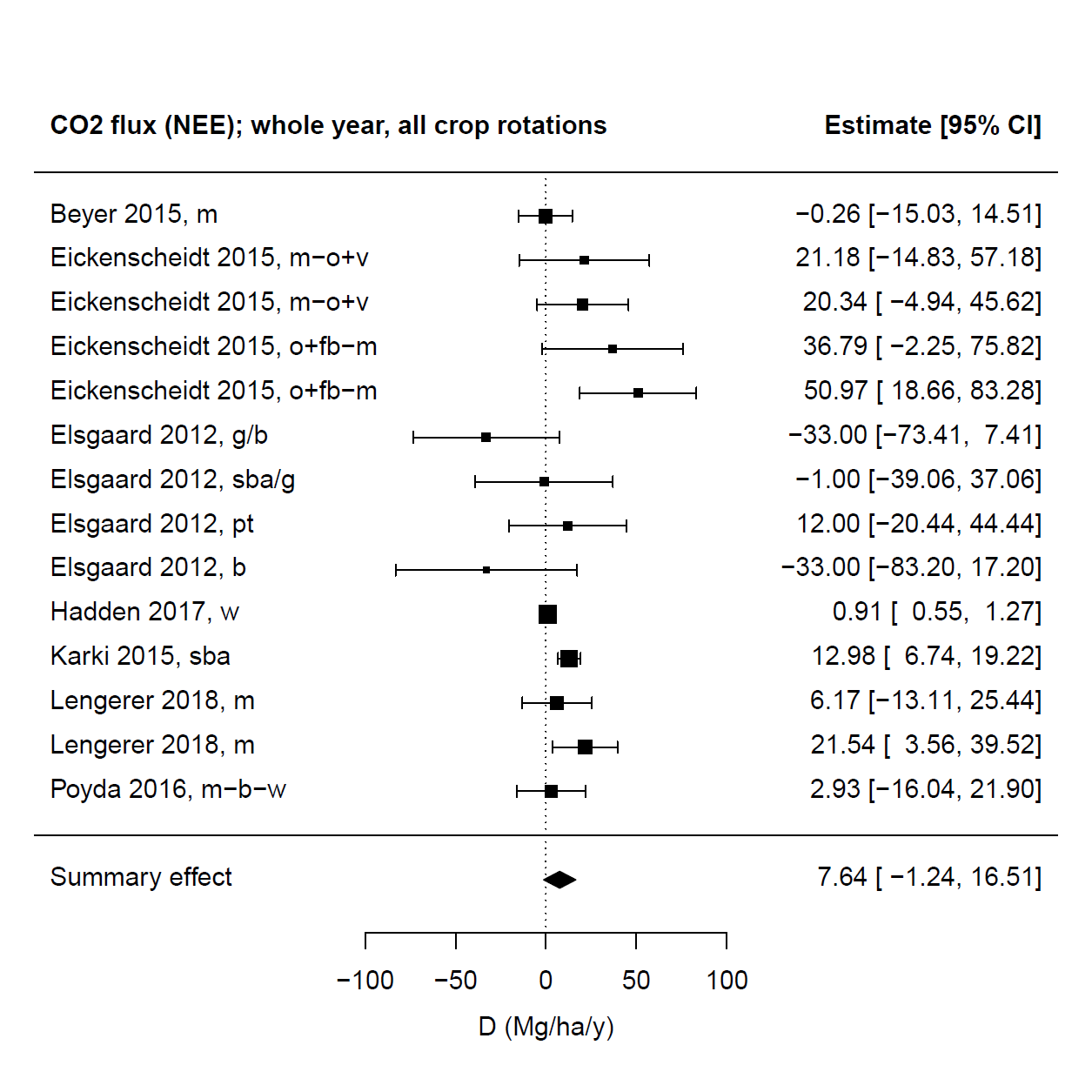

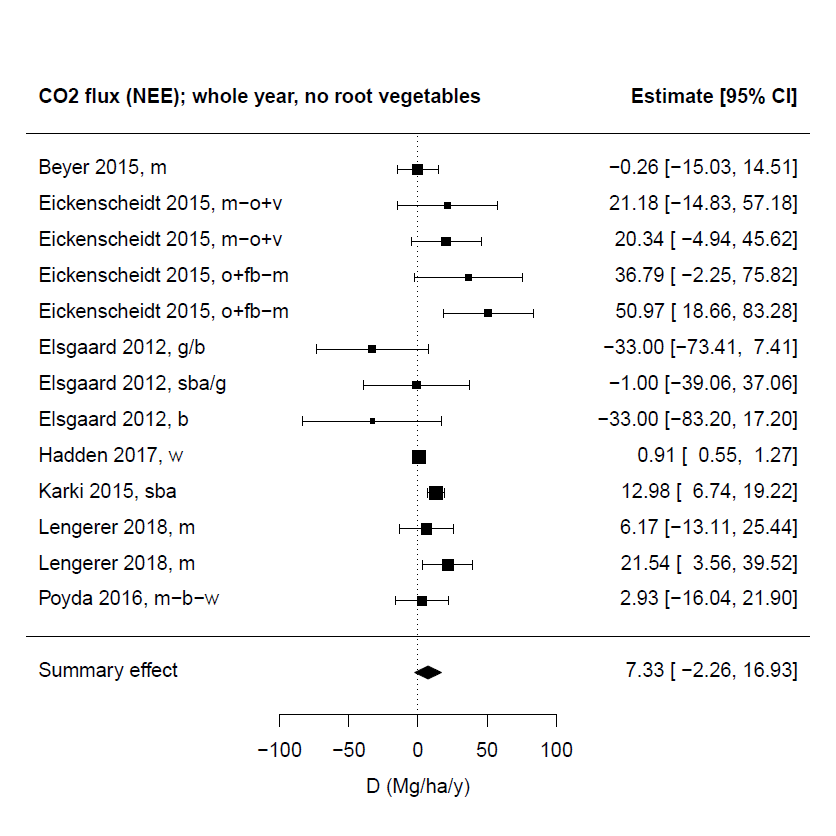


**Figure S1.** Forest plots showing raw mean differences in NEE (D_NEE_) between grasslands and croplands. To the left are all crop rotations included in the comparator group, to the right are crop rotations involving root crops excluded.


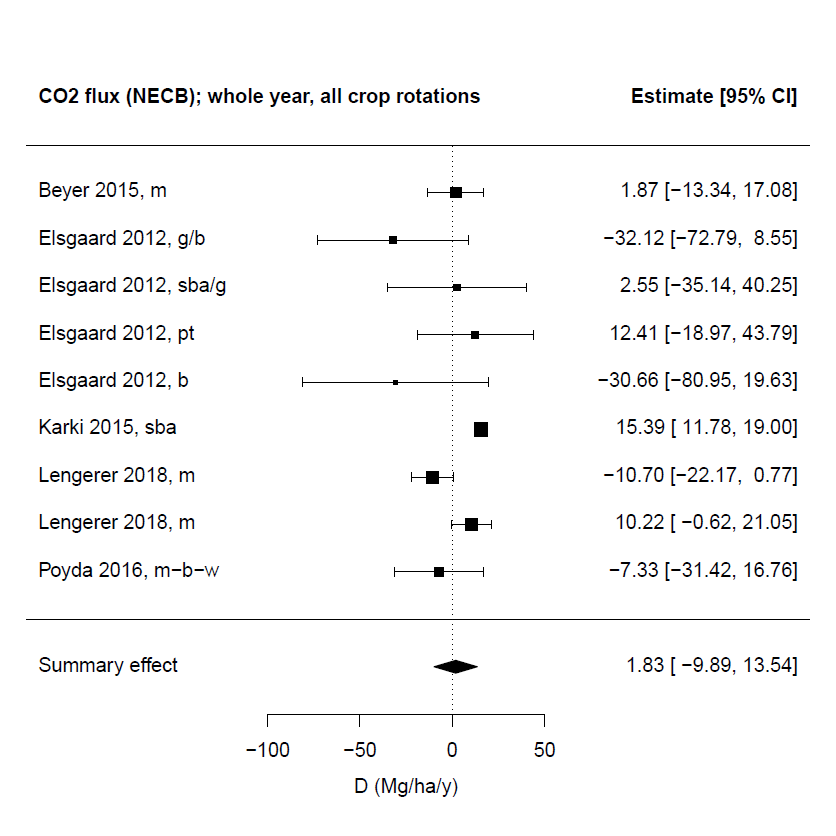

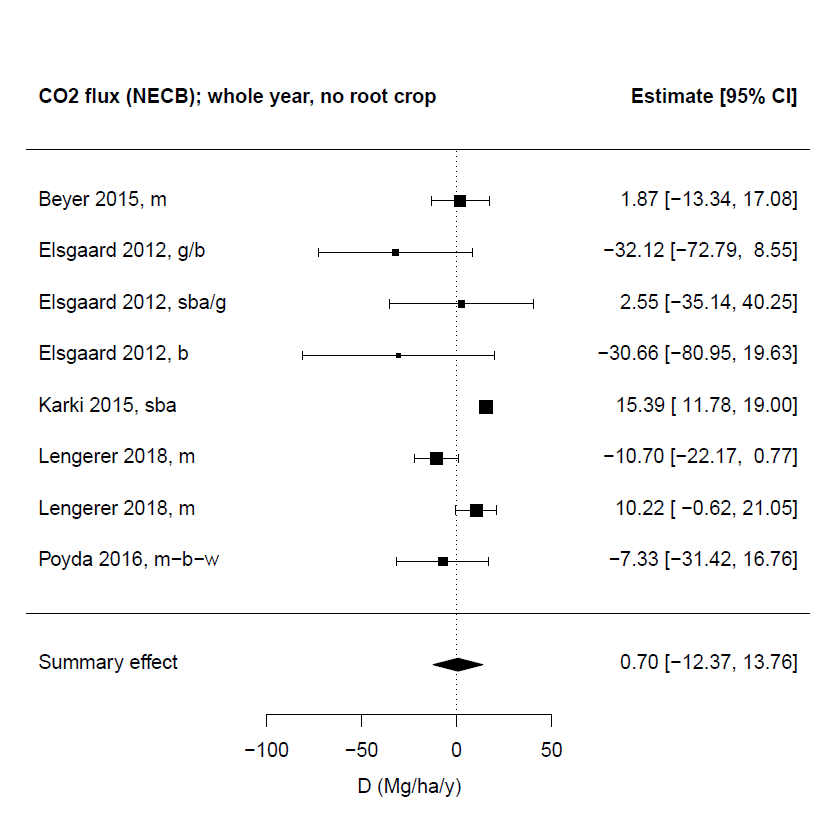


**Figure S2.** Forest plots showing raw mean differences in NECB (D_NECB_) between grasslands and croplands. To the left are all crop rotations included in the comparator group, to the right are crop rotations involving root crops excluded.


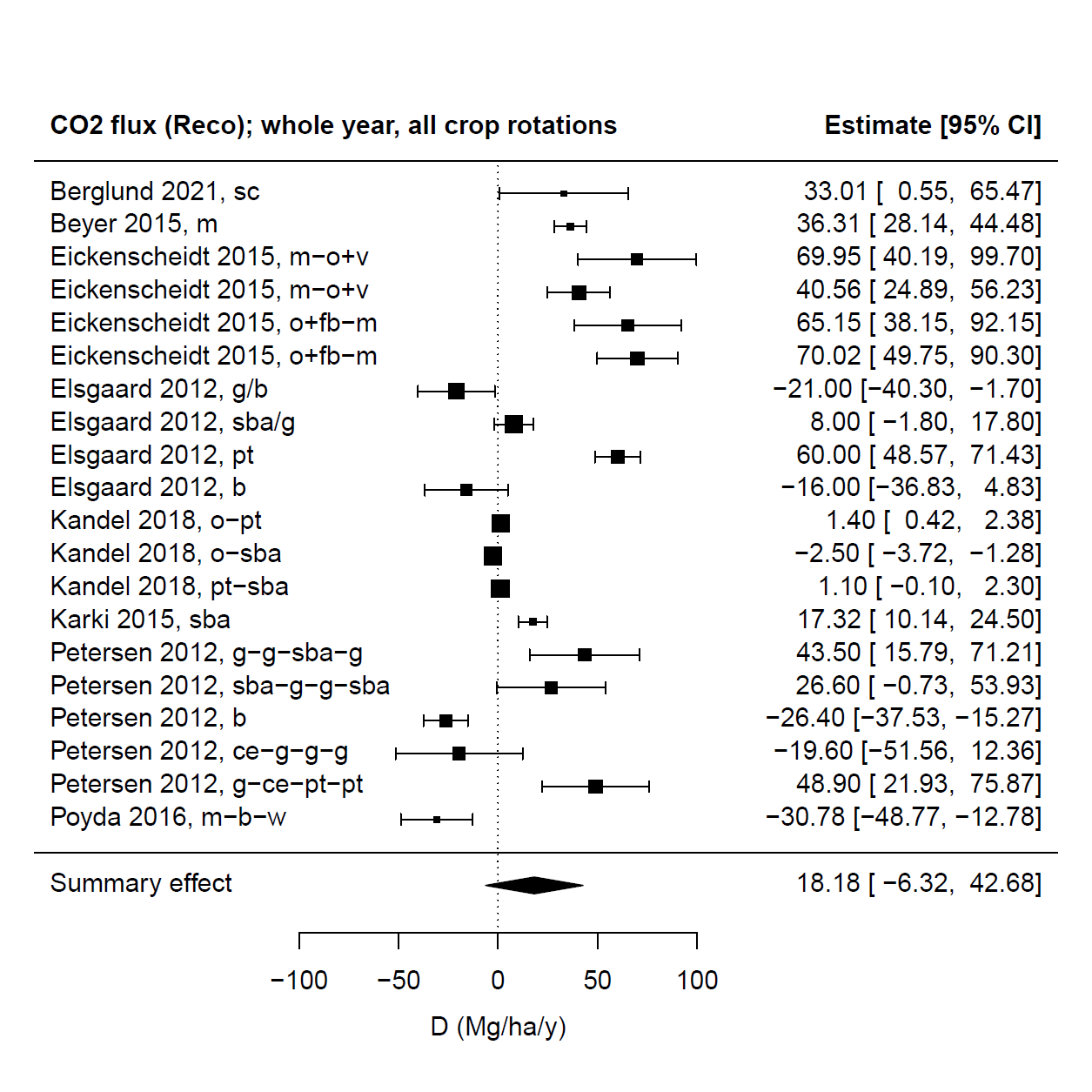

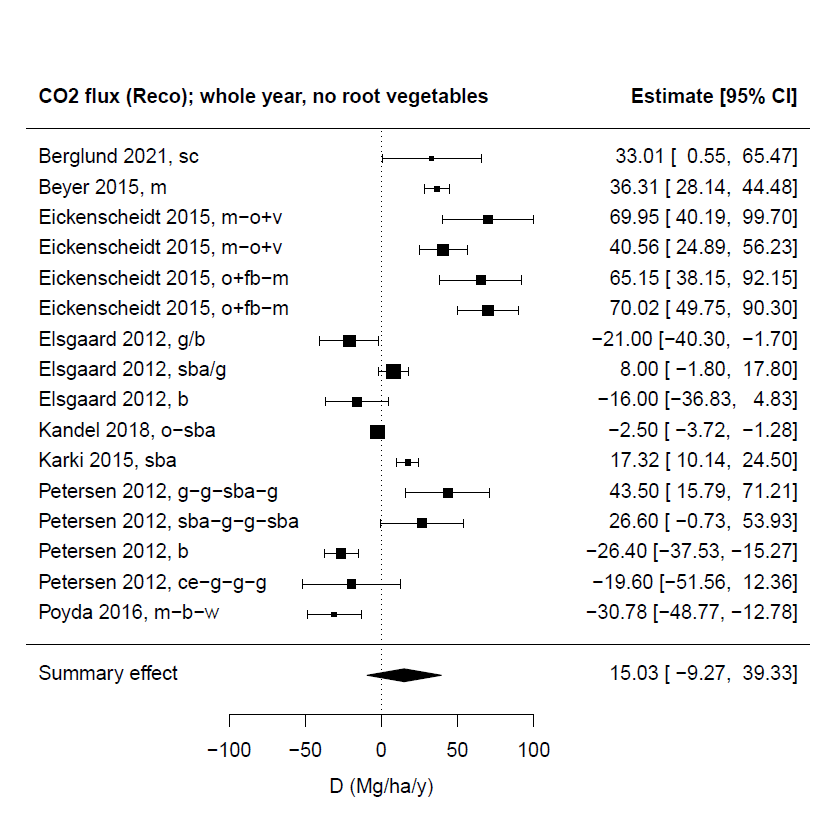


**Figure S3.** Forest plots showing raw mean differences in R_eco_ (D_Reco_) between grasslands and croplands. To the left are all crop rotations included in the comparator group, to the right are crop rotations involving root crops excluded.


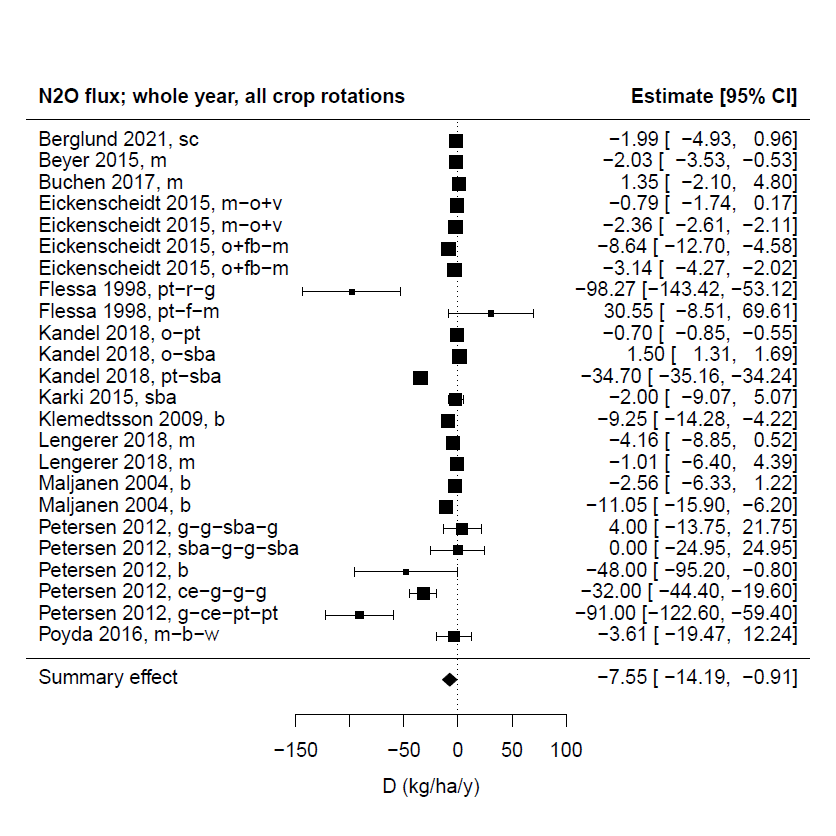

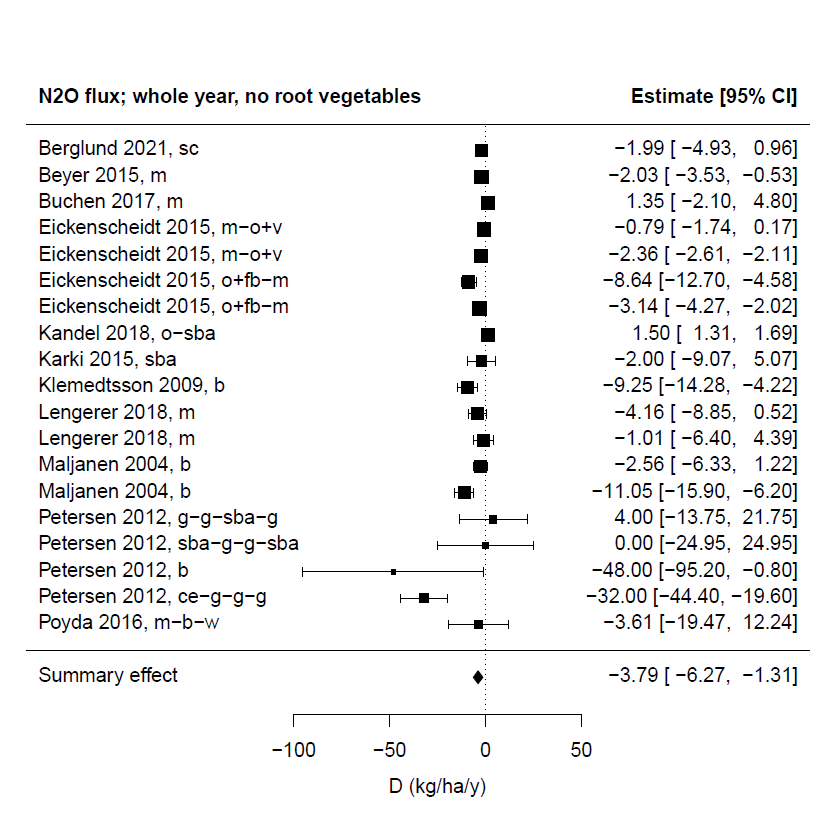


**Figure S4.** Forest plots showing raw mean differences in N_2_O flux (D_N2O_) between grasslands and croplands. To the left are all crop rotations included in the comparator group, to the right are crop rotations involving root crops excluded.


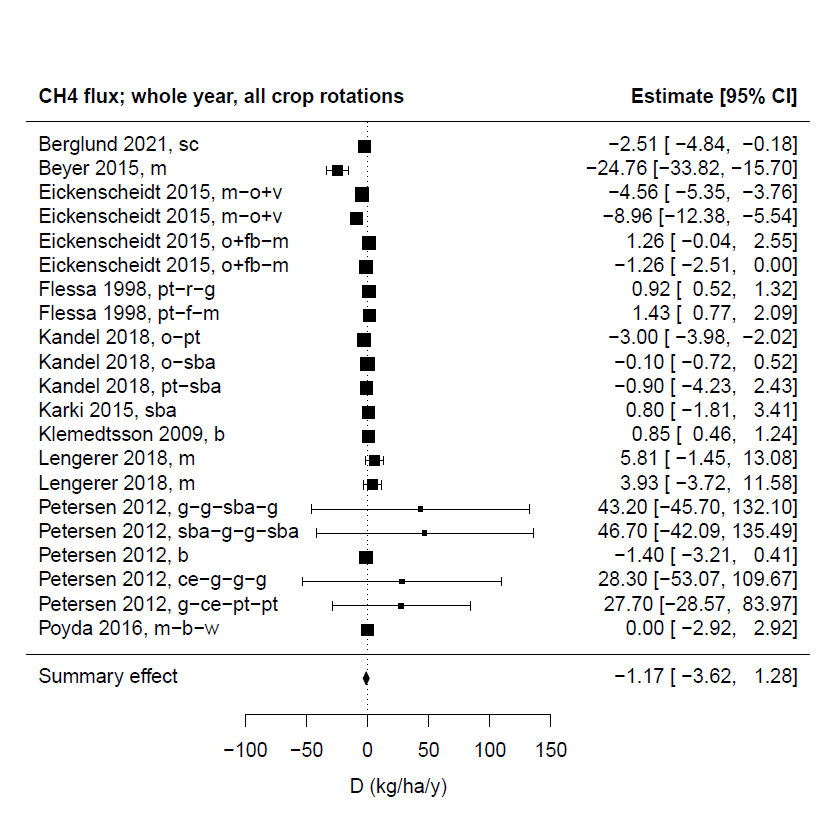

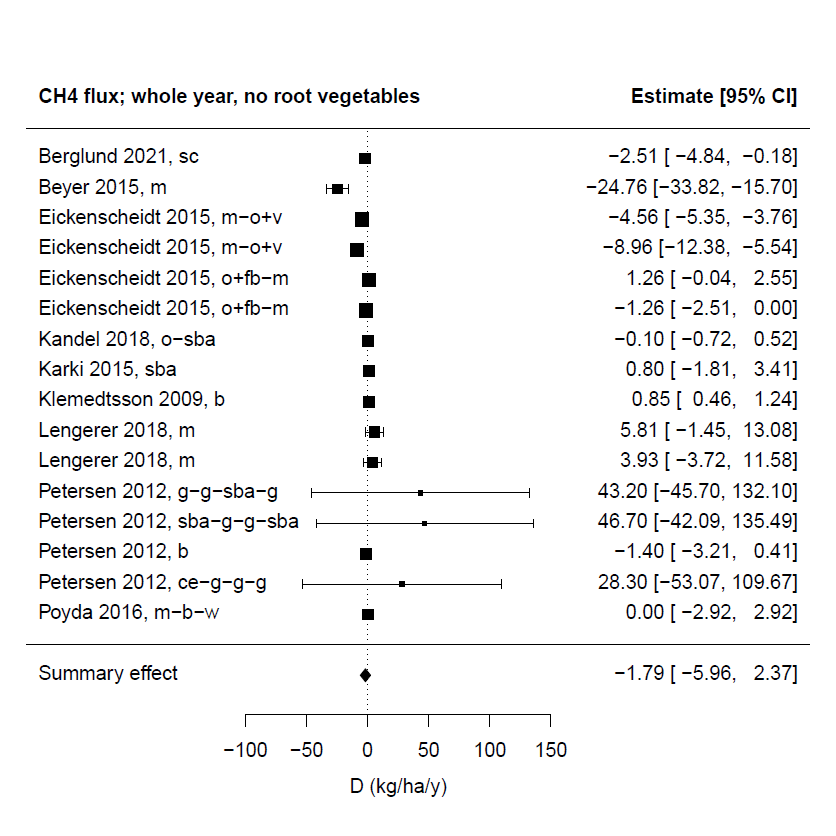


**Figure S5.** Forest plots showing raw mean differences in CH_4_ flux (D_CH4_) between grasslands and croplands. To the left are all crop rotations included in the comparator group, to the right are crop rotations involving root crops excluded.

## Meta-regression model with one categorical moderator

**Table S2.** Compilation of results from meta-analyses using a model with a single categorical moderator and intercept. Including intercept in the model means that the first subgroup within a certain moderator is used as reference, while the results shown for the following subgroups should be interpreted as the difference between the subgroup and the reference subgroup. Subgroup analysis is made for moderators with at least two subgroups and subgroups with at least two comparisons (k). D is raw mean difference and se is standard error.

| **Response** | **Moderator** | **Subgroup** | **D** | **se** | **p-value** | **k** |
| --- | --- | --- | --- | --- | --- | --- |
| Reco | Climate zone | Cfb | 16.5046 | 12.9846 | 0.219876 | 19 |
| N2O | Climate zone | Cfb | -8.58968 | 4.27015 | 0.0572742 | 21 |
| N2O | Climate zone | Dfc | 1.81157 | 12.938 | 0.889978 | 2 |
| CH4 | Climate zone | Cfb | -1.12931 | 1.51257 | 0.464432 | 20 |
| NEE | Grassland type | intensive grassland | 16.1672 | 4.36802 | **0.00349384** | 7 |
| NEE | Grassland type | low intensity grassland | -16.7989 | 7.2294 | **0.040315** | 6 |
| Reco | Grassland type | intensive grassland | 18.3002 | 21.9851 | 0.41744 | 6 |
| Reco | Grassland type | low intensity grassland | -2.47537 | 28.7994 | 0.932571 | 10 |
| Reco | Grassland type | pasture | -7.13228 | 31.1732 | 0.821927 | 3 |
| NECB | Grassland type | intensive grassland | 11.3366 | 4.38174 | **0.0360898** | 3 |
| NECB | Grassland type | low intensity grassland | -16.7119 | 6.92206 | **0.0464798** | 6 |
| N2O | Grassland type | intensive grassland | -9.3581 | 7.15398 | 0.205676 | 12 |
| N2O | Grassland type | low intensity grassland | -3.22795 | 10.7273 | 0.76659 | 8 |
| N2O | Grassland type | pasture | 20.1475 | 15.1578 | 0.198753 | 3 |
| CH4 | Grassland type | intensive grassland | -0.323294 | 2.53553 | 0.900037 | 9 |
| CH4 | Grassland type | low intensity grassland | -2.29965 | 3.88413 | 0.561602 | 8 |
| CH4 | Grassland type | pasture | -2.10306 | 6.17253 | 0.737494 | 3 |
| NEE | Land use conversion | cropping to grassland | 13.4181 | 7.27798 | 0.0983373 | 6 |
| NEE | Land use conversion | unclear | -17.8949 | 11.9854 | 0.169624 | 5 |
| Reco | Land use conversion | cropping to grassland | 39.0991 | 17.582 | **0.0400028** | 6 |
| Reco | Land use conversion | grassland to cropping | -39.3645 | 23.7881 | 0.11631 | 4 |
| Reco | Land use conversion | unclear | -28.783 | 22.1886 | 0.211893 | 10 |
| NECB | Land use conversion | unclear | -17.3779 | 12.1838 | 0.226932 | 5 |
| N2O | Land use conversion | cropping to grassland | -2.76346 | 10.8792 | 0.802364 | 6 |
| N2O | Land use conversion | grassland to cropping | 3.47886 | 14.23 | 0.809629 | 6 |
| N2O | Land use conversion | old conversion | -26.24 | 23.6423 | 0.28166 | 2 |
| N2O | Land use conversion | unclear | -15.7636 | 14.112 | 0.278668 | 8 |
| CH4 | Land use conversion | cropping to grassland | -2.08256 | 2.12412 | 0.342423 | 6 |
| CH4 | Land use conversion | grassland to cropping | 1.53522 | 3.04892 | 0.621905 | 5 |
| CH4 | Land use conversion | old conversion | 3.25877 | 3.66303 | 0.387706 | 2 |
| CH4 | Land use conversion | unclear | -5.84533 | 4.10041 | 0.174477 | 6 |
| NEE | Comparator type | cereal | 8.9198 | 5.20378 | 0.114511 | 12 |
| Reco | Comparator type | cereal | 15.2618 | 11.7363 | 0.20987 | 16 |
| Reco | Comparator type | root crop | 21.0558 | 10.2542 | 0.0548589 | 4 |
| NECB | Comparator type | cereal | 0.69714 | 5.52596 | 0.903155 | 8 |
| N2O | Comparator type | cereal | -4.33073 | 2.48461 | 0.095291 | 19 |
| N2O | Comparator type | root crop | -21.3461 | 6.5141 | **0.00344561** | 5 |
| CH4 | Comparator type | cereal | -1.5029 | 1.42198 | 0.303808 | 16 |
| CH4 | Comparator type | root crop | 1.09011 | 2.55873 | 0.67487 | 5 |

## Meta-regression model with one continuous moderator

**Table S3.** Compilation of results from meta-regressions using a multilevel random effects model with a single continuous moderator. Number of comparisons is denoted by k.

| **Response** | **Moderator** | **Coefficient** | **p-value** | **k** |
| --- | --- | --- | --- | --- |
| NEE | Grassland SOC (%) | -0.11748 | 0.80733 | 14 |
| Reco | Grassland SOC (%) | 1.20931 | 0.14205 | 20 |
| NECB | Grassland SOC (%) | 1.59462 | **0.0307** | 9 |
| N2O | Grassland SOC (%) | -0.43955 | 0.13984 | 24 |
| CH4 | Grassland SOC (%) | 0.07048 | 0.59156 | 21 |
| NEE | Grassland total N (%) | 6.09183 | 0.34819 | 14 |
| Reco | Grassland total N (%) | 11.03957 | 0.44039 | 20 |
| NECB | Grassland total N (%) | 12.13673 | 0.0614 | 9 |
| N2O | Grassland total N (%) | 0.00021 | 0.89637 | 24 |
| CH4 | Grassland total N (%) | 2.61808 | 0.06673 | 21 |
| NEE | Grassland soil C/N ratio | -2.01589 | **0.00865** | 14 |
| Reco | Grassland soil C/N ratio | 1.24729 | 0.46215 | 20 |
| NECB | Grassland soil C/N ratio | -0.42464 | 0.68913 | 9 |
| N2O | Grassland soil C/N ratio | -0.59757 | 0.18854 | 24 |
| CH4 | Grassland soil C/N ratio | -0.29389 | 0.46251 | 21 |
| NEE | Grassland soil pH | -1.03247 | 0.88087 | 13 |
| Reco | Grassland soil pH | 7.43023 | 0.59321 | 20 |
| NECB | Grassland soil pH | 11.16922 | 0.18255 | 9 |
| N2O | Grassland soil pH | 0.70107 | 0.85198 | 24 |
| CH4 | Grassland soil pH | 1.34312 | 0.15904 | 21 |
| NEE | Grassland soil bulk density | 32.73036 | 0.32088 | 13 |
| Reco | Grassland soil bulk density | -10.82258 | 0.7795 | 15 |
| NECB | Grassland soil bulk density | -89.23543 | 0.14815 | 9 |
| N2O | Grassland soil bulk density | 10.3201 | 0.41657 | 19 |
| CH4 | Grassland soil bulk density | -6.61378 | 0.49952 | 16 |
| NEE | Grassland N fertilization (kg/ha/y) | 0.20856 | **0.00044** | 13 |
| Reco | Grassland N fertilization (kg/ha/y) | 0.07352 | 0.79689 | 19 |
| NECB | Grassland N fertilization (kg/ha/y) | 0.17676 | 0.40466 | 8 |
| N2O | Grassland N fertilization (kg/ha/y) | 0.0424 | 0.50006 | 22 |
| CH4 | Grassland N fertilization (kg/ha/y) | -0.01696 | 0.67199 | 19 |
| NEE | Cropland SOC (%) | -0.14803 | 0.73574 | 14 |
| Reco | Cropland SOC (%) | 1.60996 | **0.02512** | 20 |
| NECB | Cropland SOC (%) | 0.76683 | 0.24481 | 9 |
| N2O | Cropland SOC (%) | -0.53674 | 0.09924 | 24 |
| CH4 | Cropland SOC (%) | 0.13151 | 0.16063 | 21 |
| NEE | Cropland total N (%) | 3.20871 | 0.60069 | 14 |
| Reco | Cropland total N (%) | 16.45201 | 0.32569 | 20 |
| NECB | Cropland total N (%) | 8.46408 | 0.2178 | 9 |
| N2O | Cropland total N (%) | 0.00027 | 0.85656 | 24 |
| CH4 | Cropland total N (%) | 2.65556 | **0.03111** | 21 |
| NEE | Cropland soil C/N ratio | -1.28773 | 0.08832 | 14 |
| Reco | Cropland soil C/N ratio | 2.84543 | 0.07344 | 20 |
| NECB | Cropland soil C/N ratio | -0.21207 | 0.82279 | 9 |
| N2O | Cropland soil C/N ratio | -0.5734 | 0.17484 | 24 |
| CH4 | Cropland soil C/N ratio | -0.24503 | 0.45979 | 21 |
| NEE | Cropland soil pH | -1.11796 | 0.89807 | 13 |
| Reco | Cropland soil pH | -10.8876 | 0.2173 | 20 |
| NECB | Cropland soil pH | 5.61904 | 0.52681 | 9 |
| N2O | Cropland soil pH | 9.18874 | **0.0238** | 24 |
| CH4 | Cropland soil pH | 0.79737 | 0.61543 | 21 |
| NEE | Cropland soil bulk density | 34.30455 | 0.32674 | 13 |
| Reco | Cropland soil bulk density | 12.72644 | 0.80147 | 15 |
| NECB | Cropland soil bulk density | -45.93809 | 0.34151 | 9 |
| N2O | Cropland soil bulk density | 11.22367 | 0.39671 | 19 |
| CH4 | Cropland soil bulk density | -7.33792 | 0.46242 | 16 |
| NEE | Cropland N fertilization (kg/ha/y) | 0.04397 | 0.81722 | 10 |
| Reco | Cropland N fertilization (kg/ha/y) | -0.0088 | 0.95381 | 15 |
| NECB | Cropland N fertilization (kg/ha/y) | 0.18318 | 0.29094 | 6 |
| N2O | Cropland N fertilization (kg/ha/y) | 0.13463 | 0.20241 | 17 |
| CH4 | Cropland N fertilization (kg/ha/y) | 0.01004 | 0.32371 | 14 |
| NEE | Difference in SOC (%) | 0.64795 | 0.60164 | 13 |
| Reco | Difference in SOC (%) | -1.72426 | 0.23957 | 19 |
| NECB | Difference in SOC (%) | 0.56475 | 0.65449 | 8 |
| N2O | Difference in SOC (%) | 1.6728 | 0.11296 | 22 |
| CH4 | Difference in SOC (%) | -0.31684 | 0.40326 | 20 |
| NEE | Difference in total N (%) | 21.3278 | 0.23323 | 13 |
| Reco | Difference in total N (%) | -2.26988 | 0.93522 | 19 |
| NECB | Difference in total N (%) | 23.82234 | 0.0747 | 8 |
| N2O | Difference in total N (%) | -0.00519 | 0.66052 | 22 |
| CH4 | Difference in total N (%) | -3.40236 | 0.45744 | 20 |
| NEE | Difference in soil C/N ratio | -4.89988 | 0.2722 | 13 |
| Reco | Difference in soil C/N ratio | -7.29392 | **0.04611** | 19 |
| NECB | Difference in soil C/N ratio | -8.36737 | 0.12119 | 8 |
| N2O | Difference in soil C/N ratio | -1.64796 | 0.50223 | 22 |
| CH4 | Difference in soil C/N ratio | 0.38556 | 0.73655 | 20 |
| NEE | Difference in soil pH | 0.51799 | 0.96318 | 12 |
| Reco | Difference in soil pH | 17.44507 | **0.04854** | 19 |
| NECB | Difference in soil pH | 19.89888 | 0.20171 | 8 |
| N2O | Difference in soil pH | -15.03957 | **0.02278** | 22 |
| CH4 | Difference in soil pH | 1.68364 | 0.43225 | 20 |
| NEE | Difference in soil bulk density | -55.32201 | 0.58609 | 12 |
| Reco | Difference in soil bulk density | -118.03088 | 0.26171 | 14 |
| NECB | Difference in soil bulk density | -98.24629 | 0.31707 | 8 |
| N2O | Difference in soil bulk density | -9.26519 | 0.8446 | 17 |
| CH4 | Difference in soil bulk density | -7.46476 | 0.81121 | 15 |
| NEE | Difference in N fertilization (kg/ha/y) | 0.26556 | **0.04238** | 9 |
| Reco | Difference in N fertilization (kg/ha/y) | 0.0086 | 0.95286 | 14 |
| NECB | Difference in N fertilization (kg/ha/y) | 0.05136 | 0.98804 | 5 |
| N2O | Difference in N fertilization (kg/ha/y) | -0.09268 | 0.34058 | 15 |
| CH4 | Difference in N fertilization (kg/ha/y) | -0.00845 | 0.22838 | 13 |
| NEE | Average SOC (%) | -0.13659 | 0.76988 | 14 |
| Reco | Average SOC (%) | 1.53982 | 0.05476 | 20 |
| NECB | Average SOC (%) | 1.1201 | 0.12391 | 9 |
| N2O | Average SOC (%) | -0.49812 | 0.11476 | 24 |
| CH4 | Average SOC (%) | 0.10386 | 0.42856 | 21 |
| NEE | Average total N (%) | 4.75583 | 0.45909 | 14 |
| Reco | Average total N (%) | 14.41514 | 0.36845 | 20 |
| NECB | Average total N (%) | 10.39764 | 0.12836 | 9 |
| N2O | Average total N (%) | 0.00025 | 0.87521 | 24 |
| CH4 | Average total N (%) | 2.78953 | **0.03795** | 21 |
| NEE | Average soil C/N ratio | -1.60731 | **0.03881** | 14 |
| Reco | Average soil C/N ratio | 2.09764 | 0.21223 | 20 |
| NECB | Average soil C/N ratio | -0.3096 | 0.7578 | 9 |
| N2O | Average soil C/N ratio | -0.58908 | 0.18194 | 24 |
| CH4 | Average soil C/N ratio | -0.27393 | 0.45553 | 21 |
| NEE | Average soil pH | -1.33485 | 0.87357 | 13 |
| Reco | Average soil pH | -7.51633 | 0.56116 | 20 |
| NECB | Average soil pH | 9.42639 | 0.28663 | 9 |
| N2O | Average soil pH | 5.05739 | 0.22109 | 24 |
| CH4 | Average soil pH | 1.22896 | 0.44123 | 21 |
| NEE | Average soil bulk density | 34.3387 | 0.3215 | 13 |
| Reco | Average soil bulk density | -2.52309 | 0.95479 | 15 |
| NECB | Average soil bulk density | -68.20789 | 0.22263 | 9 |
| N2O | Average soil bulk density | 11.01643 | 0.40108 | 19 |
| CH4 | Average soil bulk density | -7.18965 | 0.47466 | 16 |
| NEE | Average N fertilization (kg/ha/y) | 0.1647 | **0.04677** | 13 |
| Reco | Average N fertilization (kg/ha/y) | 0.02055 | 0.93182 | 19 |
| NECB | Average N fertilization (kg/ha/y) | 0.20074 | 0.13979 | 8 |
| N2O | Average N fertilization (kg/ha/y) | 0.0803 | 0.38855 | 22 |
| CH4 | Average N fertilization (kg/ha/y) | 0.02105 | 0.57683 | 19 |

### Regression plots

Below are regression plots shown for significant results (p<0.05) in Table S3.

**b)**

| 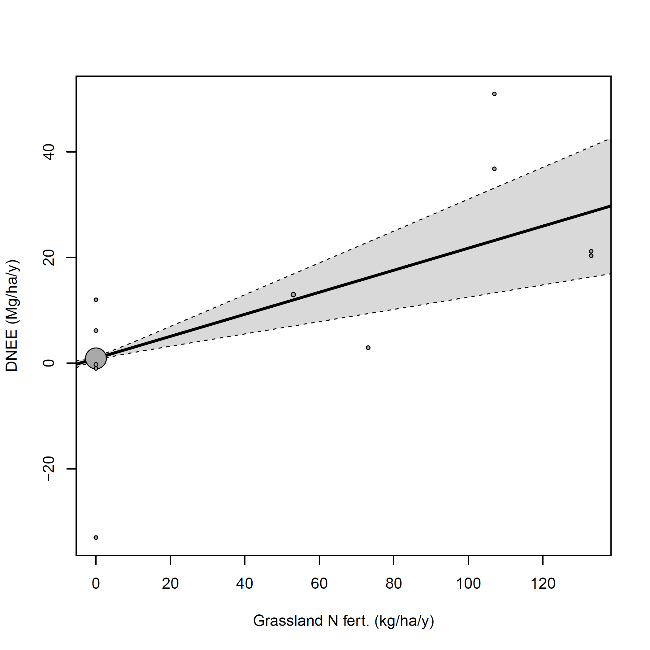  **a)** | 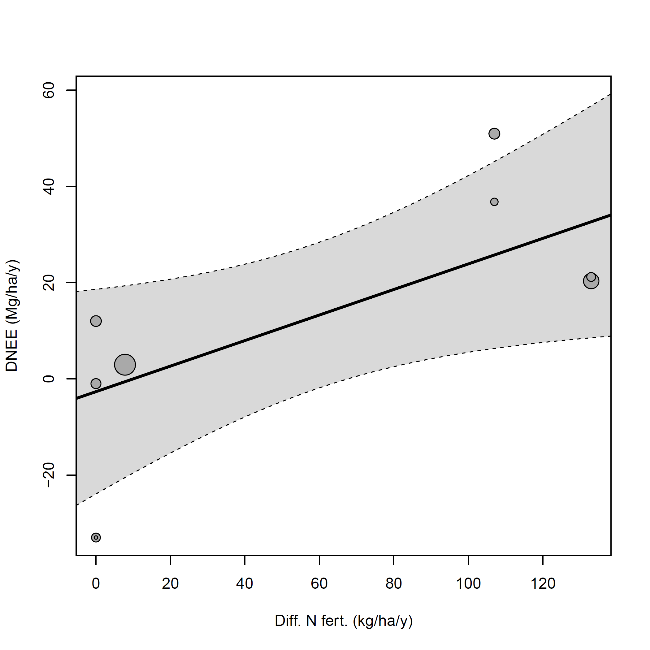  **d)** |
| --- | --- |
| 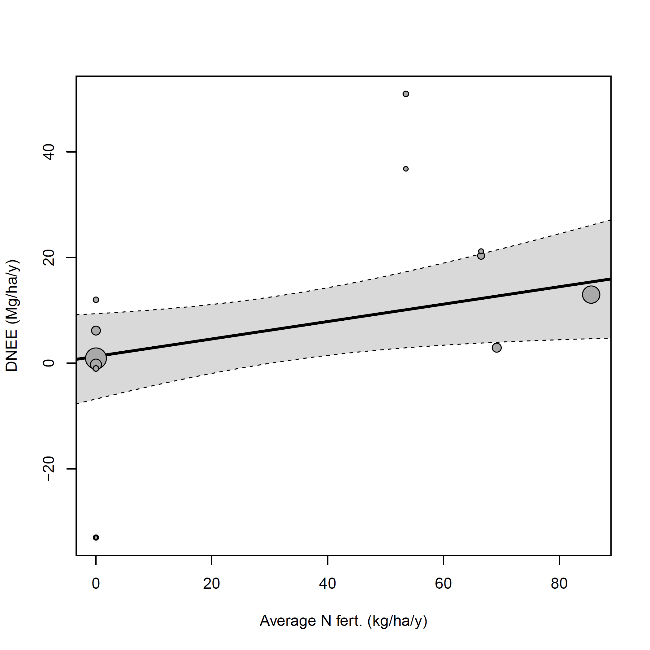  **c)** | 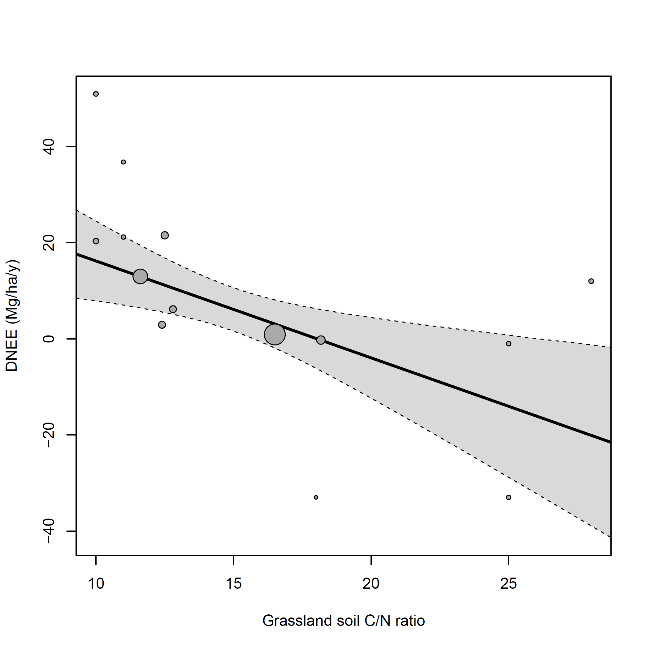 |
| 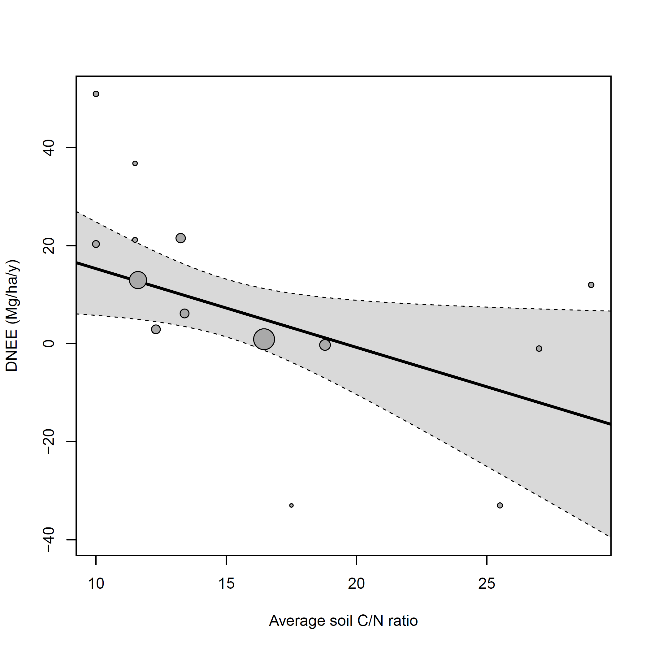  **e)** | **Figure S6.** Regression plots showing significant (p<0.05) correlations between D_NEE_ and **a)** Grassland N fertilization, **b)** difference in N fertilization between grasslands and croplands, **c)** average N fertilisation rate on grasslands and croplands, **d)** soil C/N ratio on grassland, and **e)** average soil C/N ratio. All crop rotations are included in the comparator group. |


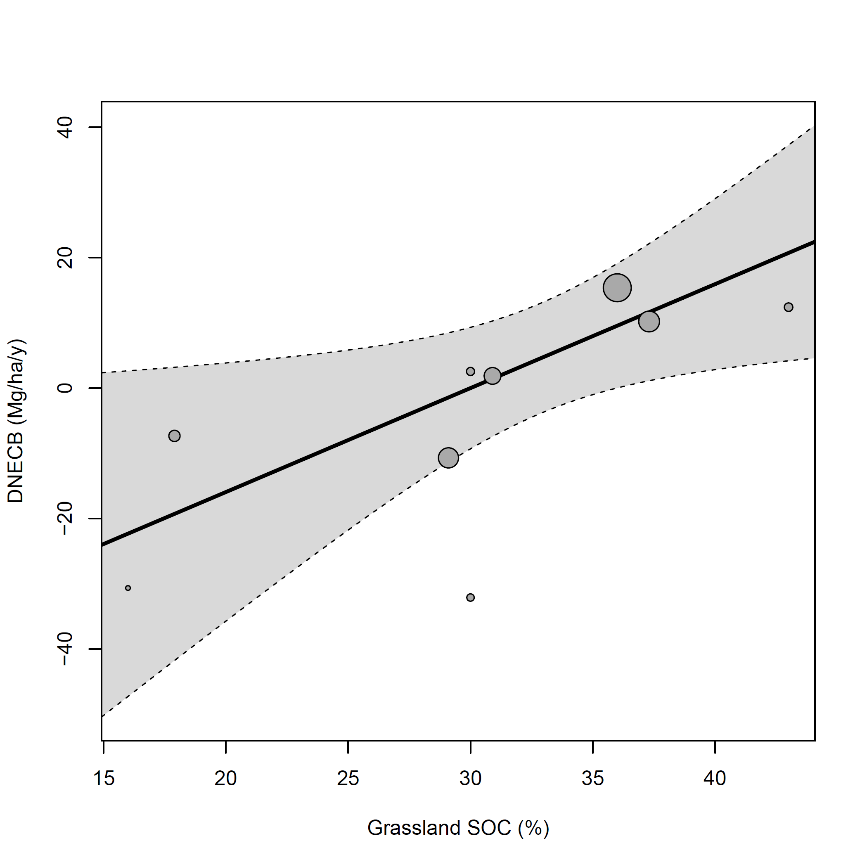


**Figure S7.** Regression plot showing correlations between D_NECB_ and Grassland SOC concentration. All crop rotations are included in the comparator group.


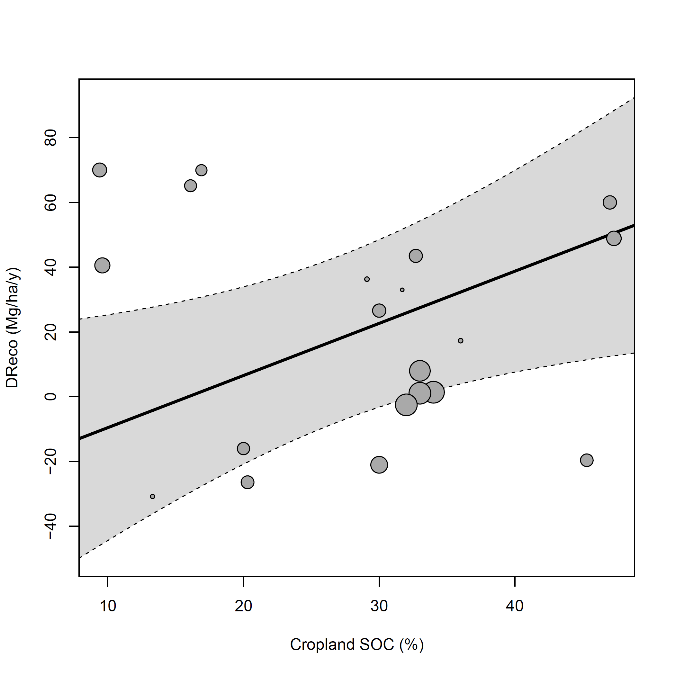

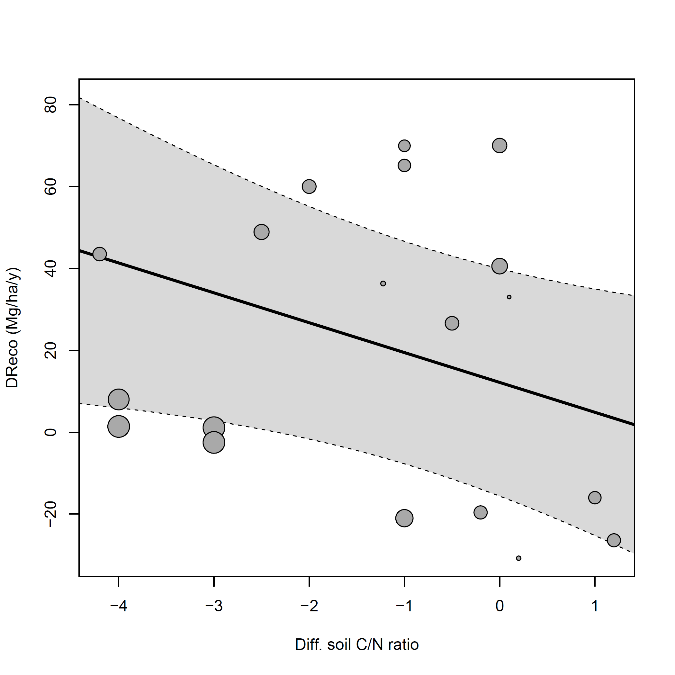


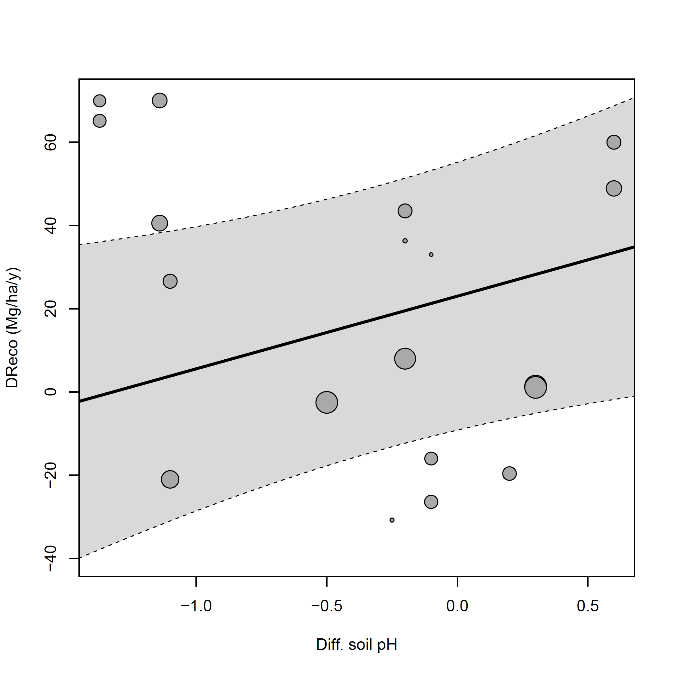


**b)**

**c)**

**a)**

**Figure S8.** Regression plots showing correlations between D_Reco_ and **a)** cropland SOC concentration, **b)** difference in soil C/N ratio between grasslands and croplands, and **c)** difference in soil pH between grasslands and croplands. All crop rotations are included in the comparator group.


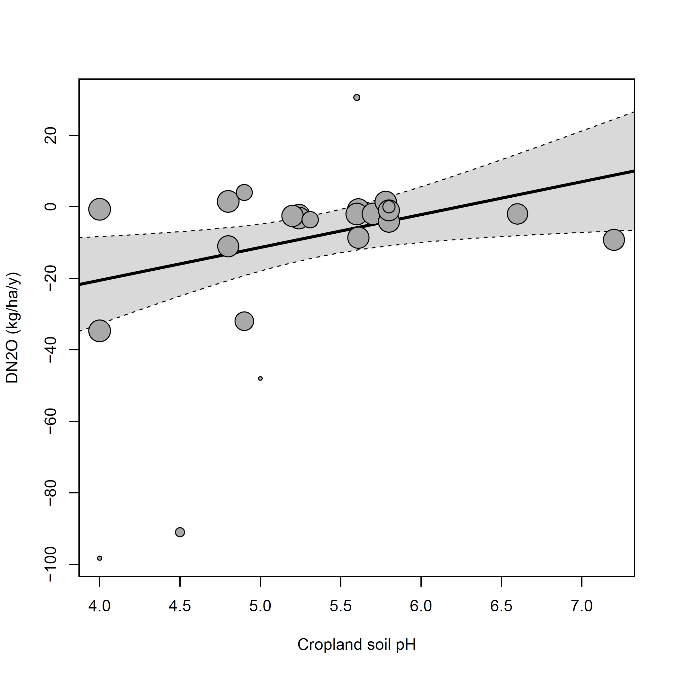

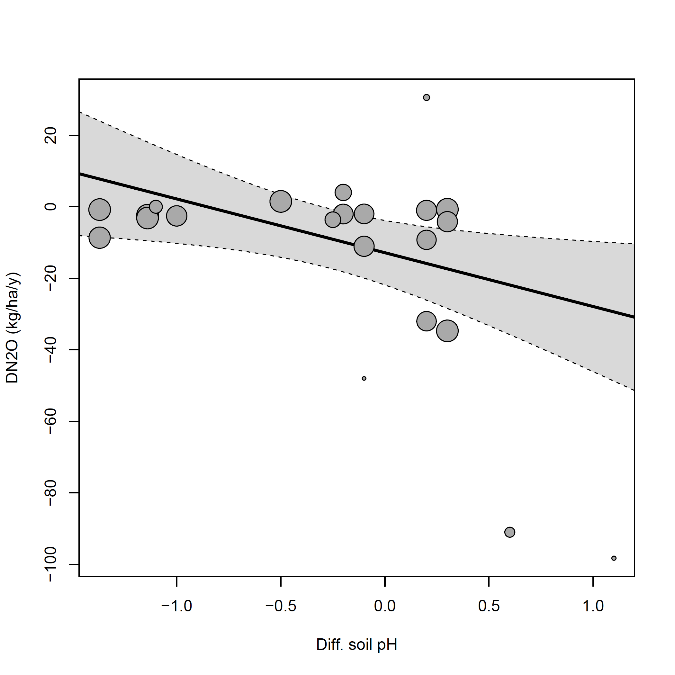


**b)**

**a)**

**Figure S9.** Regression plots showing correlations between D_N2O_ and **a)** cropland soil pH and **b)** difference in soil pH between grasslands and croplands. All crop rotations are included in the comparator group.


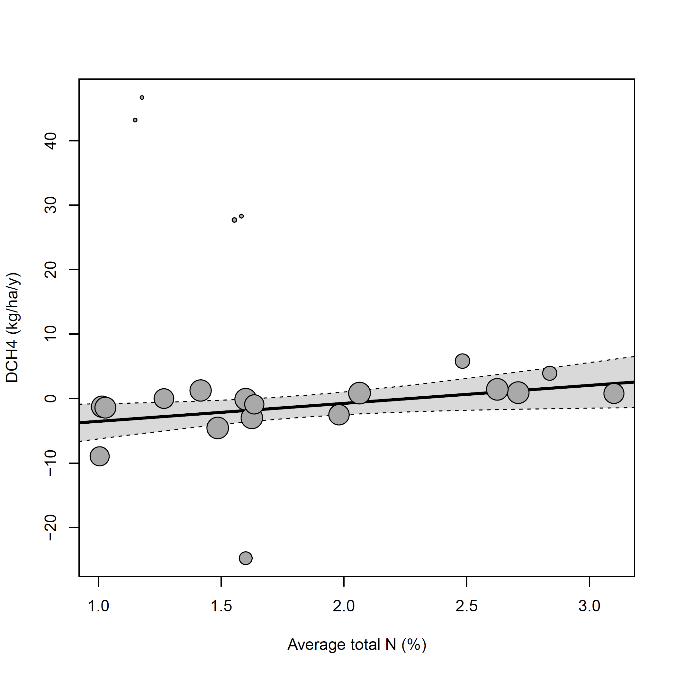

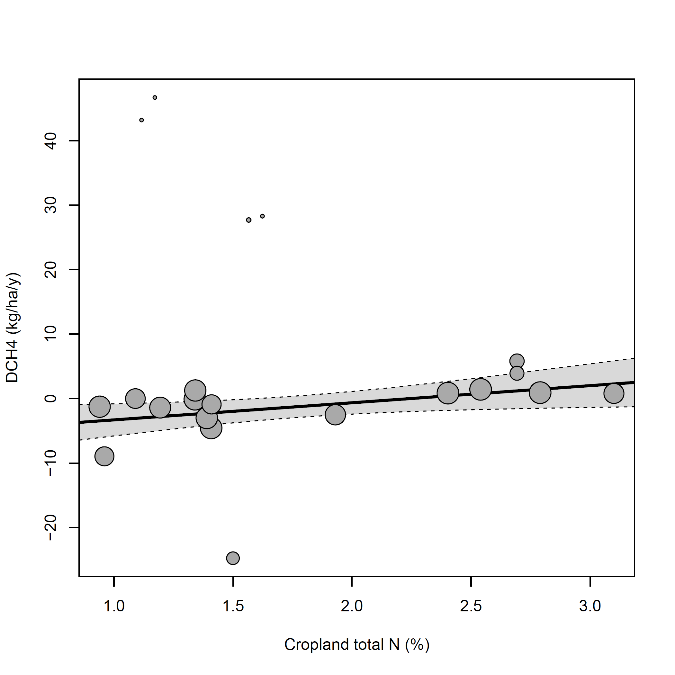


**b)**

**a)**

**Figure S10.** Regression plots showing correlations between D_CH4_ and **a)** average soil total N concentration and **b)** cropland soil total N concentration. All crop rotations are included in the comparator group.

## Risk of bias Sensitivity Analysis

**Table S4.** Compilation of results from meta-analyses using intercept-only models and including only studies with low risk of bias. D is raw mean difference (=X_grassland_ – X_cropland_ where X is mean flux), k is number of comparisons, and I^2^_tot_ and I^2^_loc_ is total heterogeneity and heterogeneity among study locations, respectively.

| **Season** | **Comparator** | **Flux** | **Summary D [95% CI]** | **p-value** | **k** | **I2 tot (%)** | **I2 loc (%)** |
| --- | --- | --- | --- | --- | --- | --- | --- |
| whole year | all crop rotations | NEE (Mg/ha/y) | 7.64 [-1.24, 16.5] | 0.0858 | 14 | 68 | 68 |
| whole year | all crop rotations | Reco (Mg/ha/y) | 18.2 [-6.32, 42.7] | 0.137 | 20 | 100 | 78 |
| whole year | all crop rotations | NECB (Mg/ha/y) | 1.83 [-9.89, 13.5] | 0.728 | 9 | 70 | 0.00000057 |
| whole year | all crop rotations | N2O (kg/ha/y) | -6.42 [-12.5, -0.316] | **0.0402** | 21 | 100 | 13 |
| whole year | all crop rotations | CH4 (kg/ha/y) | -2.38 [-7.23, 2.47] | 0.315 | 18 | 98 | 75 |

**Table S5.** Compilation of results from meta-analyses using a model with a single categorical moderator and intercept, including only studies with a low risk of bias. Including intercept in the model means that the first subgroup for a certain moderator is used as reference, while the results shown for the following subgroups should be interpreted as the difference between the subgroup and the reference subgroup. Subgroup analysis is made for moderators with at least two subgroups and subgroups with at least two comparisons (k). D is raw mean difference and se is standard error.

| **Response** | **Moderator** | **Subgroup** | **D** | **se** | **p-value** | **k** |
| --- | --- | --- | --- | --- | --- | --- |
| Reco | Climate zone | Cfb | 16.5046 | 12.9846 | 0.219876 | 19 |
| N2O | Climate zone | Cfb | -6.87934 | 3.70789 | 0.0800025 | 18 |
| N2O | Climate zone | Dfc | 0.1132 | 10.4818 | 0.991502 | 2 |
| CH4 | Climate zone | Cfb | -2.48799 | 2.86082 | 0.397335 | 17 |
| NEE | Grassland type | intensive grassland | 16.1672 | 4.36802 | **0.00349384** | 7 |
| NEE | Grassland type | low intensity grassland | -16.7989 | 7.2294 | **0.040315** | 6 |
| Reco | Grassland type | intensive grassland | 18.3002 | 21.9851 | 0.41744 | 6 |
| Reco | Grassland type | low intensity grassland | -2.47537 | 28.7994 | 0.932571 | 10 |
| Reco | Grassland type | pasture | -7.13228 | 31.1732 | 0.821927 | 3 |
| NECB | Grassland type | intensive grassland | 11.3366 | 4.38174 | **0.0360898** | 3 |
| NECB | Grassland type | low intensity grassland | -16.7119 | 6.92206 | **0.0464798** | 6 |
| N2O | Grassland type | intensive grassland | -3.45472 | 5.04497 | 0.50271 | 10 |
| N2O | Grassland type | low intensity grassland | -11.8102 | 8.2684 | 0.171306 | 7 |
| N2O | Grassland type | pasture | 0.108721 | 10.5563 | 0.991903 | 3 |
| CH4 | Grassland type | intensive grassland | -1.62983 | 4.24224 | 0.706609 | 7 |
| CH4 | Grassland type | low intensity grassland | -1.77994 | 5.32534 | 0.743153 | 7 |
| CH4 | Grassland type | pasture | -2.21894 | 9.1846 | 0.812598 | 3 |
| NEE | Land use conversion | cropping to grassland | 13.4181 | 7.27798 | 0.0983373 | 6 |
| NEE | Land use conversion | unclear | -17.8949 | 11.9854 | 0.169624 | 5 |
| Reco | Land use conversion | cropping to grassland | 39.0991 | 17.582 | **0.0400028** | 6 |
| Reco | Land use conversion | grassland to cropping | -39.3645 | 23.7881 | 0.11631 | 4 |
| Reco | Land use conversion | unclear | -28.783 | 22.1886 | 0.211893 | 10 |
| NECB | Land use conversion | unclear | -17.3779 | 12.1838 | 0.226932 | 5 |
| N2O | Land use conversion | cropping to grassland | -2.86394 | 7.58638 | 0.710754 | 6 |
| N2O | Land use conversion | grassland to cropping | 2.25538 | 10.7629 | 0.836662 | 5 |
| N2O | Land use conversion | unclear | -12.4078 | 10.2312 | 0.242832 | 8 |
| CH4 | Land use conversion | cropping to grassland | -1.72497 | 3.83624 | 0.660368 | 6 |
| CH4 | Land use conversion | grassland to cropping | 0.0895492 | 6.00076 | 0.98832 | 4 |
| CH4 | Land use conversion | unclear | -7.05968 | 6.40944 | 0.290677 | 6 |
| NEE | Comparator type | cereal | 8.9198 | 5.20378 | 0.114511 | 12 |
| Reco | Comparator type | cereal | 15.2618 | 11.7363 | 0.20987 | 16 |
| Reco | Comparator type | root crop | 21.0558 | 10.2542 | 0.0548589 | 4 |
| NECB | Comparator type | cereal | 0.69714 | 5.52596 | 0.903155 | 8 |
| N2O | Comparator type | cereal | -3.91702 | 2.358 | 0.113093 | 18 |
| N2O | Comparator type | root crop | -20.67 | 6.39026 | **0.00436191** | 3 |
| CH4 | Comparator type | cereal | -2.27064 | 2.38603 | 0.355435 | 15 |
| CH4 | Comparator type | root crop | -1.22578 | 3.91562 | 0.758287 | 3 |

**Table S6.** Compilation of results from meta-regressions using a multilevel random effects model with a single continuous moderator and including only studies with a low risk of bias. Number of comparisons is denoted by k.

| **Response** | **Moderator** | **Coefficient** | **p-value** | **k** |
| --- | --- | --- | --- | --- |
| NEE | Grassland SOC (%) | -0.11748 | 0.80733 | 14 |
| Reco | Grassland SOC (%) | 1.20931 | 0.14205 | 20 |
| NECB | Grassland SOC (%) | 1.59462 | 0.0307 | 9 |
| N2O | Grassland SOC (%) | -0.32693 | 0.2335 | 21 |
| CH4 | Grassland SOC (%) | 0.08175 | 0.7298 | 18 |
| NEE | Grassland total N (%) | 6.09183 | 0.34819 | 14 |
| Reco | Grassland total N (%) | 11.03957 | 0.44039 | 20 |
| NECB | Grassland total N (%) | 12.13673 | 0.0614 | 9 |
| N2O | Grassland total N (%) | 0.00001 | 0.9934 | 21 |
| CH4 | Grassland total N (%) | 2.56363 | 0.40659 | 18 |
| NEE | Grassland soil C/N ratio | -2.01589 | 0.00865 | 14 |
| Reco | Grassland soil C/N ratio | 1.24729 | 0.46215 | 20 |
| NECB | Grassland soil C/N ratio | -0.42464 | 0.68913 | 9 |
| N2O | Grassland soil C/N ratio | -0.5818 | 0.15178 | 21 |
| CH4 | Grassland soil C/N ratio | -0.21866 | 0.74379 | 18 |
| NEE | Grassland soil pH | -1.03247 | 0.88087 | 13 |
| Reco | Grassland soil pH | 7.43023 | 0.59321 | 20 |
| NECB | Grassland soil pH | 11.16922 | 0.18255 | 9 |
| N2O | Grassland soil pH | 1.00669 | 0.79662 | 21 |
| CH4 | Grassland soil pH | 1.79097 | 0.59119 | 18 |
| NEE | Grassland soil bulk density | 32.73036 | 0.32088 | 13 |
| Reco | Grassland soil bulk density | -10.82258 | 0.7795 | 15 |
| NECB | Grassland soil bulk density | -89.23543 | 0.14815 | 9 |
| N2O | Grassland soil bulk density | 9.43622 | 0.43803 | 16 |
| CH4 | Grassland soil bulk density | -4.68201 | 0.70965 | 13 |
| NEE | Grassland N fertilization (kg/ha/y) | 0.20856 | 0.00044 | 13 |
| Reco | Grassland N fertilization (kg/ha/y) | 0.07352 | 0.79689 | 19 |
| NECB | Grassland N fertilization (kg/ha/y) | 0.17676 | 0.40466 | 8 |
| N2O | Grassland N fertilization (kg/ha/y) | 0.07558 | 0.13169 | 19 |
| CH4 | Grassland N fertilization (kg/ha/y) | -0.12709 | 0.0775 | 16 |
| NEE | Cropland SOC (%) | -0.14803 | 0.73574 | 14 |
| Reco | Cropland SOC (%) | 1.60996 | 0.02512 | 20 |
| NECB | Cropland SOC (%) | 0.76683 | 0.24481 | 9 |
| N2O | Cropland SOC (%) | -0.3849 | 0.17878 | 21 |
| CH4 | Cropland SOC (%) | 0.15939 | 0.50457 | 18 |
| NEE | Cropland total N (%) | 3.20871 | 0.60069 | 14 |
| Reco | Cropland total N (%) | 16.45201 | 0.32569 | 20 |
| NECB | Cropland total N (%) | 8.46408 | 0.2178 | 9 |
| N2O | Cropland total N (%) | 0.00009 | 0.94389 | 21 |
| CH4 | Cropland total N (%) | 3.32523 | 0.29259 | 18 |
| NEE | Cropland soil C/N ratio | -1.28773 | 0.08832 | 14 |
| Reco | Cropland soil C/N ratio | 2.84543 | 0.07344 | 20 |
| NECB | Cropland soil C/N ratio | -0.21207 | 0.82279 | 9 |
| N2O | Cropland soil C/N ratio | -0.62177 | 0.10285 | 21 |
| CH4 | Cropland soil C/N ratio | -0.1474 | 0.78486 | 18 |
| NEE | Cropland soil pH | -1.11796 | 0.89807 | 13 |
| Reco | Cropland soil pH | -10.8876 | 0.2173 | 20 |
| NECB | Cropland soil pH | 5.61904 | 0.52681 | 9 |
| N2O | Cropland soil pH | 9.13095 | 0.02064 | 21 |
| CH4 | Cropland soil pH | 2.07123 | 0.486 | 18 |
| NEE | Cropland soil bulk density | 34.30455 | 0.32674 | 13 |
| Reco | Cropland soil bulk density | 12.72644 | 0.80147 | 15 |
| NECB | Cropland soil bulk density | -45.93809 | 0.34151 | 9 |
| N2O | Cropland soil bulk density | 10.52184 | 0.41208 | 16 |
| CH4 | Cropland soil bulk density | -3.25132 | 0.82292 | 13 |
| NEE | Cropland N fertilization (kg/ha/y) | 0.04397 | 0.81722 | 10 |
| Reco | Cropland N fertilization (kg/ha/y) | -0.0088 | 0.95381 | 15 |
| NECB | Cropland N fertilization (kg/ha/y) | 0.18318 | 0.29094 | 6 |
| N2O | Cropland N fertilization (kg/ha/y) | -0.06419 | 0.54798 | 14 |
| CH4 | Cropland N fertilization (kg/ha/y) | 0.02031 | 0.32944 | 11 |
| NEE | Difference in SOC (%) | 0.64795 | 0.60164 | 13 |
| Reco | Difference in SOC (%) | -1.72426 | 0.23957 | 19 |
| NECB | Difference in SOC (%) | 0.56475 | 0.65449 | 8 |
| N2O | Difference in SOC (%) | 1.50273 | 0.12444 | 19 |
| CH4 | Difference in SOC (%) | -0.36813 | 0.50072 | 17 |
| NEE | Difference in total N (%) | 21.3278 | 0.23323 | 13 |
| Reco | Difference in total N (%) | -2.26988 | 0.93522 | 19 |
| NECB | Difference in total N (%) | 23.82234 | 0.0747 | 8 |
| N2O | Difference in total N (%) | -0.00433 | 0.64863 | 19 |
| CH4 | Difference in total N (%) | -2.94499 | 0.696 | 17 |
| NEE | Difference in soil C/N ratio | -4.89988 | 0.2722 | 13 |
| Reco | Difference in soil C/N ratio | -7.29392 | 0.04611 | 19 |
| NECB | Difference in soil C/N ratio | -8.36737 | 0.12119 | 8 |
| N2O | Difference in soil C/N ratio | 0.12399 | 0.94965 | 19 |
| CH4 | Difference in soil C/N ratio | -0.06736 | 0.96779 | 17 |
| NEE | Difference in soil pH | 0.51799 | 0.96318 | 12 |
| Reco | Difference in soil pH | 17.44507 | 0.04854 | 19 |
| NECB | Difference in soil pH | 19.89888 | 0.20171 | 8 |
| N2O | Difference in soil pH | -9.98345 | 0.07447 | 19 |
| CH4 | Difference in soil pH | -1.24652 | 0.74792 | 17 |
| NEE | Difference in soil bulk density | -55.32201 | 0.58609 | 12 |
| Reco | Difference in soil bulk density | -118.03088 | 0.26171 | 14 |
| NECB | Difference in soil bulk density | -98.24629 | 0.31707 | 8 |
| N2O | Difference in soil bulk density | -11.99628 | 0.80013 | 14 |
| CH4 | Difference in soil bulk density | -26.21555 | 0.47687 | 12 |
| NEE | Difference in N fertilization (kg/ha/y) | 0.26556 | 0.04238 | 9 |
| Reco | Difference in N fertilization (kg/ha/y) | 0.0086 | 0.95286 | 14 |
| NECB | Difference in N fertilization (kg/ha/y) | 0.05136 | 0.98804 | 5 |
| N2O | Difference in N fertilization (kg/ha/y) | 0.09002 | 0.10957 | 12 |
| CH4 | Difference in N fertilization (kg/ha/y) | -0.00996 | 0.38053 | 10 |
| NEE | Average SOC (%) | -0.13659 | 0.76988 | 14 |
| Reco | Average SOC (%) | 1.53982 | 0.05476 | 20 |
| NECB | Average SOC (%) | 1.1201 | 0.12391 | 9 |
| N2O | Average SOC (%) | -0.36469 | 0.1996 | 21 |
| CH4 | Average SOC (%) | 0.12719 | 0.60326 | 18 |
| NEE | Average total N (%) | 4.75583 | 0.45909 | 14 |
| Reco | Average total N (%) | 14.41514 | 0.36845 | 20 |
| NECB | Average total N (%) | 10.39764 | 0.12836 | 9 |
| N2O | Average total N (%) | 0.00005 | 0.96729 | 21 |
| CH4 | Average total N (%) | 3.09027 | 0.33436 | 18 |
| NEE | Average soil C/N ratio | -1.60731 | 0.03881 | 14 |
| Reco | Average soil C/N ratio | 2.09764 | 0.21223 | 20 |
| NECB | Average soil C/N ratio | -0.3096 | 0.7578 | 9 |
| N2O | Average soil C/N ratio | -0.60719 | 0.12633 | 21 |
| CH4 | Average soil C/N ratio | -0.18199 | 0.76392 | 18 |
| NEE | Average soil pH | -1.33485 | 0.87357 | 13 |
| Reco | Average soil pH | -7.51633 | 0.56116 | 20 |
| NECB | Average soil pH | 9.42639 | 0.28663 | 9 |
| N2O | Average soil pH | 5.71921 | 0.1725 | 21 |
| CH4 | Average soil pH | 2.46294 | 0.48214 | 18 |
| NEE | Average soil bulk density | 34.3387 | 0.3215 | 13 |
| Reco | Average soil bulk density | -2.52309 | 0.95479 | 15 |
| NECB | Average soil bulk density | -68.20789 | 0.22263 | 9 |
| N2O | Average soil bulk density | 10.1765 | 0.42014 | 16 |
| CH4 | Average soil bulk density | -4.27949 | 0.75396 | 13 |
| NEE | Average N fertilization (kg/ha/y) | 0.1647 | 0.04677 | 13 |
| Reco | Average N fertilization (kg/ha/y) | 0.02055 | 0.93182 | 19 |
| NECB | Average N fertilization (kg/ha/y) | 0.20074 | 0.13979 | 8 |
| N2O | Average N fertilization (kg/ha/y) | 0.03166 | 0.70313 | 19 |
| CH4 | Average N fertilization (kg/ha/y) | -0.02081 | 0.80726 | 16 |

## Leave-one-out analysis

**Table S7.** Compilation of results for D_N2O_ (using the intercept-only model) when leaving one included paper out at a time. Omitting Maljanen 2004 makes the result non-significant (p>0.05).

| **Omitted article** | **D_N2O_** | **se** | **p** | **k** |
| --- | --- | --- | --- | --- |
| Berglund 2021 | -8.16191 | 3.582041 | 0.032757 | 23 |
| Beyer 2015 | -8.16465 | 3.585495 | 0.032856 | 23 |
| Buchen 2017 | -8.32102 | 3.501794 | 0.026620 | 23 |
| Eickenscheidt 2015 | -9.99887 | 4.753696 | 0.048984 | 20 |
| Flessa 1998 | -6.54542 | 2.690761 | 0.024020 | 22 |
| Kandel 2018 | -5.27848 | 2.062093 | 0.018679 | 21 |
| Karki 2015 | -8.13081 | 3.565541 | 0.032631 | 23 |
| Klemedtsson 2009 | -7.68140 | 3.606902 | 0.044633 | 23 |
| Lengerer 2018 | -8.73993 | 3.949384 | 0.038106 | 22 |
| Maljanen 2004 | -8.19080 | 3.991761 | **0.052853** | 22 |
| Petersen 2012 | -5.47239 | 2.295942 | 0.028370 | 19 |
| Poyda 2016 | -7.94136 | 3.511094 | 0.033933 | 23 |

## Funnel plots


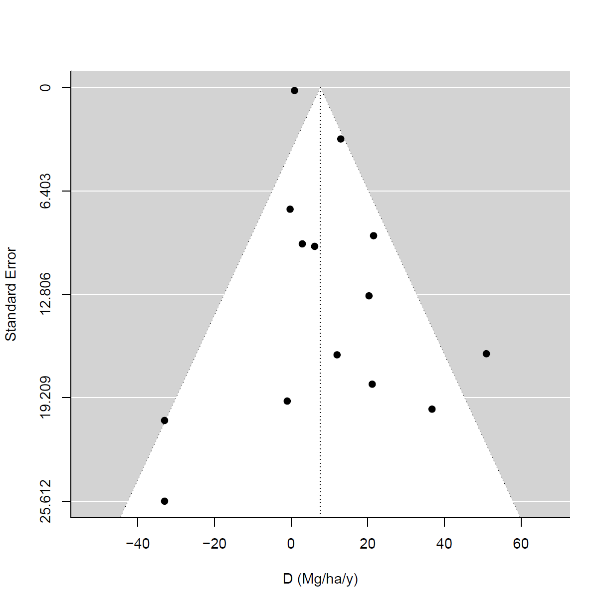

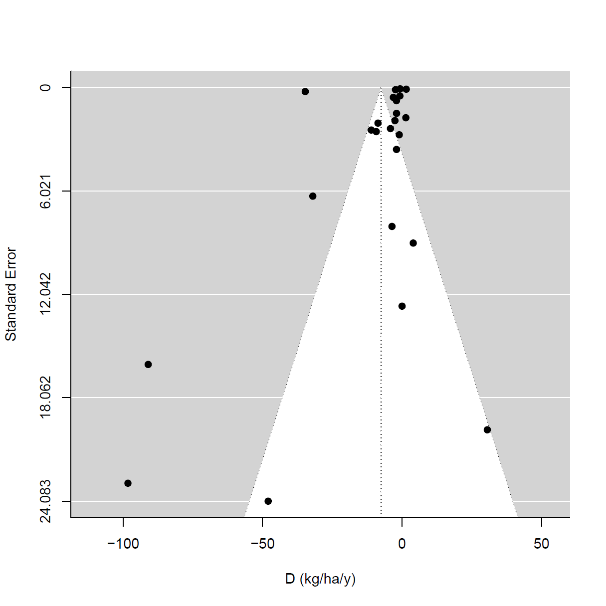

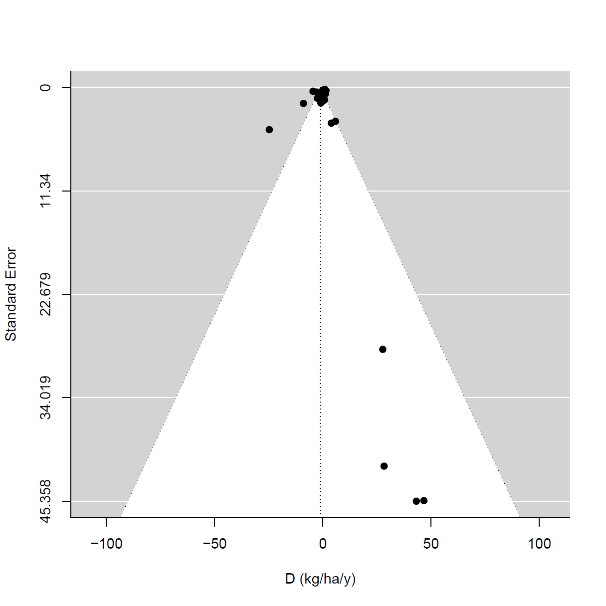

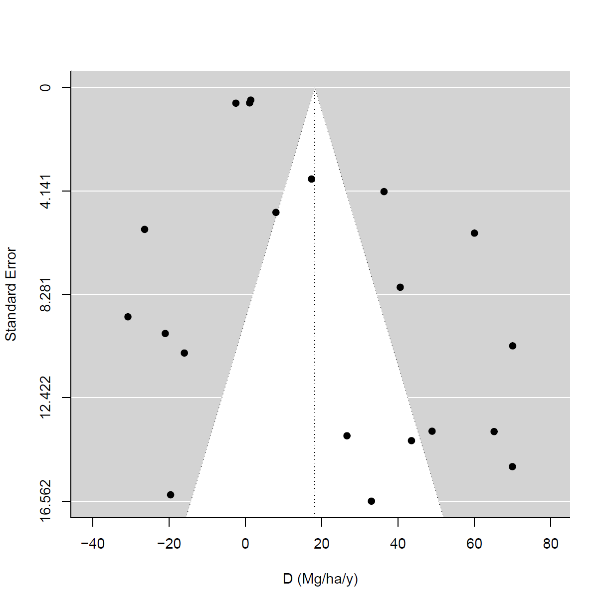

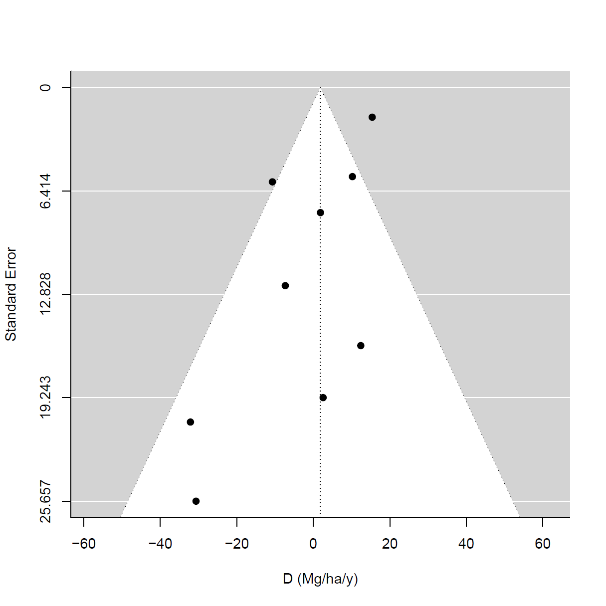


NECB (p=0.1153)

R_eco_ (p=0.7304)

CH_4_ (p=0.7702)

N_2_O (p=0.0026)

NEE (p=0.9930)

**Figure S11.** Funnel plots for assessment of risk of publication bias. The plots are based on whole-year data including all crop types in the control group. Egger’s regression test indicates a significant asymmetry for D_N2O_ (p<0.05).
